# Genome-Based Characterization of Biological Processes That Differentiate Closely Related Bacteria

**DOI:** 10.3389/fmicb.2018.00113

**Published:** 2018-02-06

**Authors:** Marike Palmer, Emma T. Steenkamp, Martin P. A. Coetzee, Jochen Blom, Stephanus N. Venter

**Affiliations:** ^1^Department of Microbiology and Plant Pathology, Forestry and Agricultural Biotechnology Institute, University of Pretoria, Pretoria, South Africa; ^2^Department of Genetic, Forestry and Agricultural Biotechnology Institute, University of Pretoria, Pretoria, South Africa; ^3^Bioinformatics and Systems Biology, Justus-Liebig-University Giessen, Giessen, Germany

**Keywords:** genome-inferred biology, *Enterobacteriaceae*, phenotype, KEGG, bacterial systematics, *Pantoea*

## Abstract

Bacteriologists have strived toward attaining a natural classification system based on evolutionary relationships for nearly 100 years. In the early twentieth century it was accepted that a phylogeny-based system would be the most appropriate, but in the absence of molecular data, this approach proved exceedingly difficult. Subsequent technical advances and the increasing availability of genome sequencing have allowed for the generation of robust phylogenies at all taxonomic levels. In this study, we explored the possibility of linking biological characters to higher-level taxonomic groups in bacteria by making use of whole genome sequence information. For this purpose, we specifically targeted the genus *Pantoea* and its four main lineages. The shared gene sets were determined for *Pantoea*, the four lineages within the genus, as well as its sister-genus *Tatumella*. This was followed by functional characterization of the gene sets using the Kyoto Encyclopedia of Genes and Genomes (KEGG) database. In comparison to *Tatumella*, various traits involved in nutrient cycling were identified within *Pantoea*, providing evidence for increased efficacy in recycling of metabolites within the genus. Additionally, a number of traits associated with pathogenicity were identified within species often associated with opportunistic infections, with some support for adaptation toward overcoming host defenses. Some traits were also only conserved within specific lineages, potentially acquired in an ancestor to the lineage and subsequently maintained. It was also observed that the species isolated from the most diverse sources were generally the most versatile in their carbon metabolism. By investigating evolution, based on the more variable genomic regions, it may be possible to detect biologically relevant differences associated with the course of evolution and speciation.

## Introduction

Since the early twentieth century scientists have recognized the value of phylogenetic inferences in determining natural relationships between taxa, which is essential for both taxonomy and evolutionary studies (Woese, 1987). However, the move toward a more natural classification system by these early bacteriologists, based on phylogenetics, proved exceedingly difficult as traditionally used morphological traits were not variable enough to group taxa reliably (Stanier and Van Niel, 1941; Woese, 1987; Woese et al., 1990). Although this led to the use of physiological characters, some researchers already argued early on that such data would not be suitable for developing evolutionary hypotheses. They emphasized that physiological traits would generally not be phylogenetically informative as long as there were no clear understanding of their genetic basis and overall biological importance (Stanier and Van Niel, 1941). The consensus view at the time was thus that phylogenetic inferences were definitely needed for elucidating the natural relationships among bacteria, but that this would only be possible with the use of suitably informative characters (Stanier and Van Niel, 1941; Woese, 1987, 1994; Woese et al., 1990). As a result, scholars mostly abandoned the field of bacterial systematics until more reliable characters became available with the advent of nucleic acid-based molecular phylogenetics in the 1970s (Woese, 1987, 1994, 1998; Woese et al., 1990; McInerney et al., 2011).

For studying bacterial systematics, the ubiquitous 16S ribosomal RNA (16S rRNA) gene was initially the marker of choice (Hillis and Dixon, 1991; Woese, 1994, 1998; Garrity et al., 2005; Gevers et al., 2005; Konstantinidis and Tiedje, 2007). Over time, however, as the diversity of examined samples increased, it became apparent that the 16 rRNA gene sequence alone does not provide sufficient phylogenetic resolution. Therefore, more reliable approaches for phylogenetic inference were sought to obtain better resolved trees. This led to the use of multiple locus sequence analyses (MLSA) (Gevers et al., 2005; Konstantinidis and Tiedje, 2007; Glaeser and Kämpfer, 2015), ribosomal MLSA (Bennett et al., 2012) and more recently core genome phylogenies (Bennett et al., 2012; Chan et al., 2012; Rahman et al., 2015; Schwartz et al., 2015; Palmer et al., 2017). These approaches, especially those based on large numbers of core genes, provide robust evolutionary hypotheses that seems to be resilient to most known phylogenetic errors (Beukes et al., 2017; Palmer et al., 2017) and have recently formed the foundation of taxonomic decisions, particularly in problematic taxa (Zhang et al., 2011; Bennett et al., 2012; Chan et al., 2012; Richards et al., 2014; Ormeno-Orrillo et al., 2015; Rahman et al., 2015).

The next logical step after having used phylogenetics to identify taxa, particularly those above the species level, would be to assign biological characters to them. For example, if bacterial genera or the lineages within them represent natural clusters, it should be possible to identify properties that they share with one another, but that are different from those occurring in other such clusters. Previously, various standardized sets of physiological tests have been used to study phenotypic cohesion of bacterial taxa (Schubert, 1968; Gavini et al., 1989; Mergaert et al., 1993; Brady et al., 2010a, 2013), but these have been mainly developed from a clinical diagnostics perspective (Konstantinidis and Tiedje, 2005a,b; Sutcliffe, 2015). Accordingly, the characters identified by these tests have limited application outside this environment (Sutcliffe et al., 2012; Sutcliffe, 2015). Other than revealing some basic physiological capabilities, these standard phenotypic tests are incapable of capturing the countless traits encoded on bacterial genomes. In addition, the very limited set of traits analyzed rarely differentiates between taxa, as the members of a taxon can show immense physiological variability. Therefore, characters that are biologically meaningful and that potentially define or distinguish higher-level bacterial groups and taxa would thus have to be sought through other means.

For identifying biological traits that are potentially taxon-defining, whole genome sequences represent invaluable resources. A wealth of traits can be inferred from a bacterium's genome sequence by making use of bioinformatics approaches and databases built from experimental evidence. For example, metabolic and physiological networks or pathways can be inferred from gene sequences by making use of their homology to sequences in the Kyoto Encyclopedia of Genes and Genomes (KEGG) (Kanehisa et al., 2016b). Each sequence in the KEGG database have an associated KEGG Orthology (KO) term, which is in turn coupled to proteins whose functions have been experimentally verified (Kanehisa et al., 2016b). In this workflow for inferring physiological properties, the database of functionally verified protein entries is often regarded as a significant limitation. This is because functional characterization of genes occurs at a much slower pace than gene discovery, thus making it impossible to functionally annotate certain genes (Linghu et al., 2008). As a result, taxa related to the model organisms typically have a higher number genes that can be functionally annotated because of the higher similarity between their genomes (Linghu et al., 2008). Despite this limitation, the current information would still provide valuable biological knowledge, especially as the information in these databases increase.

In this study, we explored the possibility of linking biological characters to higher-level taxonomic groups in bacteria by making use of whole genome sequence information. For this purpose we used the genus *Pantoea* for which genome sequences of 21 species were available (Hong et al., 2012; Kamber et al., 2012; Wan et al., 2015; Palmer et al., 2017). These were chosen to span the diversity of the genus, which includes plant pathogens (*P. agglomerans, P. ananatis, P. stewartii* amongst others; Coutinho and Venter, 2009) and species that affect humans (*P. brenneri, P. conspicua, P. eucrina*, and *P. septica;* Walterson and Stavrinides, 2015), as well as species that have been isolated from insects, fungal fruiting bodies and environmental samples (Walterson and Stavrinides, 2015; Ma et al., 2016; Palmer et al., 2016; Rong et al., 2016). Overall the members of this genus appear to be highly adaptable to changing environments and may act opportunistically when in contact with potential eukaryotic hosts (Coutinho and Venter, 2009; De Maayer et al., 2012b, 2014; Walterson and Stavrinides, 2015). From a phylogenetic perspective, *Pantoea* and its sister taxon, *Tatumella*, are nested within the *Enterobacteriaceae* where they are closely related to *Erwinia* (Glaeser and Kämpfer, 2015; Palmer et al., 2017). *Pantoea* further separates into four well-supported lineages, *viz*. the *P. agglomerans* (containing *P. agglomerans, P. anthophila, P. brenneri, P. conspicua, P. deleyi, P. eucalypti*, and *P. vagans*), *P. ananatis* (containing *P. allii, P. ananatis, P. stewartii* ssp. *stewartii* and *P. stewartii* ssp. *indologenes*), *P. rodasii* (containing *P. rodasii, P. rwandensis*, and *Pantoea* sp. GM01) and *P. dispersa* (containing *P. dispersa, P. eucrina* and *P. wallisii*) lineages (Palmer et al., 2017). Other than a limited set of general biological traits (e.g., colony and cell morphology, respiration status, growth temperature), characters that potentially define *Pantoea* and its lineages have never been identified.

**Figure 1:**
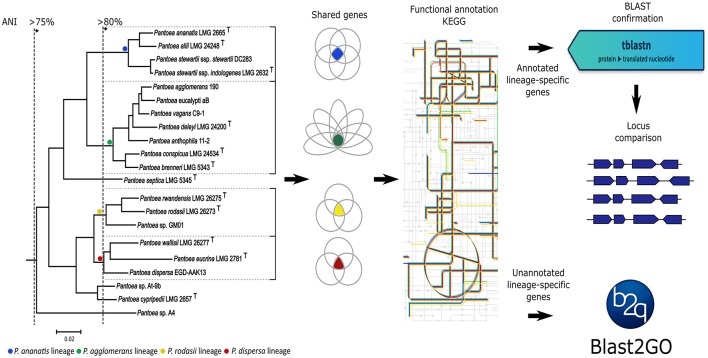
Experimental strategy followed for the lineages within *Pantoea* in the study. Lineages were identified from the subtree of *Pantoea* from the protein sequence maximum-likelihood tree of all the shared genes of Palmer et al. (2017). Average nucleotide identity (ANI) values were used as a measure of relatedness between species of a lineage as obtained from Palmer et al. (2017). Shared gene sets were determined from the genome sequences of species within each lineage. Gene sets were then annotated with the Kyoto Encyclopedia of Genes and Genomes (KEGG), followed by BLAST verification and locus comparisons of characterized genes. Uncharacterized genes were subjected to Blast2GO analyses. A similar strategy was followed for the generic comparisons with the exception of the locus comparisons.

The overall goal of this study was to link biological properties to the current evolutionary hypothesis of *Pantoea* (Palmer et al., 2017), thus allowing the identification of phenotypic characters that potentially define the genus and its lineages. Our specific aims were three-fold. First, for each of *Pantoea* and *Tatumella*, we functionally compared their shared gene sets (i.e., in terms of the pathways and processes each gene is predicted to be involved in) to evaluate the feasibility of using whole genome sequences for identifying taxon-defining characters at ranks higher than the species level. Secondly, the shared gene sets in each of the four *Pantoea* lineages were functionally compared to identify characters associated with the specific evolutionary path of the lineage and that potentially contributed to its initial emergence or subsequent maintenance. Thirdly, the loci underlying these differential characters were further characterized in order to determine their gene composition and distribution among species and whether their conservation is maintained by purifying selection as suggested before (Fang et al., 2008; Sorrels et al., 2009). Broadly, our strategy (Figure 1) involved the identification of shared gene sets, followed by their functional annotation.

## Materials and methods

### Genomes analyzed

All genomes analyzed during this study are publicly available and accessible at the National Centre for Biotechnology Information (NCBI; http://www.ncbi.nlm.nih.gov/). Whole genome sequence data for 21 species of *Pantoea* and three species of *Tatumella* (Tracz et al., 2015) were included in the analyses (Table 1). These *Pantoea* species span the current known phylogenetic and phenotypic diversity of the genus, with most representatives of all of the major lineages (Palmer et al., 2017). For the inter-generic comparisons, all 24 genomes were utilized. For the intra-generic comparisons, we included only 17 *Pantoea* genomes. We excluded three of the known lineages of this genus as one contained only two species (i.e., *Pantoea* sp. At-9b and *P. cypripedii* LMG 2655 ^T^), while the other two are each represented by single species (i.e., *Pantoea* sp. A4 and *P. septica* LMG 5345 ^T^).

**Table 1:** Genomes analyzed in the study.

**Species**	**Strain**	**Origin**	**Accession number**
*Pantoea agglomerans*	R 190	Apple, Korea	JNGC00000000.1
*Pantoea allii*	LMG 24248^T^	Onion seed, South Africa	MLFE00000000.1
*Pantoea ananatis*	LMG 2665^T^	Pineapple, Brazil	JFZU00000000.1
*Pantoea anthophila*	11-2	Hypersaline lake, Hawaii	JXXL00000000.1
*Pantoea brenneri*	LMG 5343^T^	Human, USA	MIEI00000000.1
*Pantoea calida*	LMG 25383^T^	Infant formula, -	MLFO00000000.1
*Pantoea conspicua*	LMG 24534^T^	Human, France	MLFN00000000.1
*Pantoea cypripedii*	LMG 2657^T^	Orchid, USA	MLJI00000000.1
*Pantoea deleyi*	LMG 24200^T^	Eucalyptus, Uganda	MIPO00000000.1
*Pantoea dispersa*	EGD-AAK13	Soil, India	AVSS00000000.1
*Pantoea eucalypti*	aB	Bark beetle, USA	AEDL00000000.1
*Pantoea eucrina*	LMG 2781^T^	Human, USA	MIPP00000000.1
*Pantoea gaviniae*	LMG 25382^T^	Infant formula, -	MLFQ00000000.1
*Pantoea rodasii*	LMG 26273^T^	Eucalyptus, Colombia	MLFP00000000.1
*Pantoea rwandensis*	LMG 26275^T^	Eucalyptus, Rwanda	MLFR00000000.1
*Pantoea septica*	LMG 5345^T^	Human, USA	MLJJ00000000.1
*Pantoea stewartii* ssp. *stewartii*	DC 283	Maize, USA	AHIE00000000.1
*Pantoea stewartii* ssp. *indologenes*	LMG 2632^T^	Fox millet, India	JPKO00000000.1
*Pantoea vagans*	C9-1	Apple, USA	CP001894.1, CP001893.1, CP001894.1
*Pantoea wallisii*	LMG 26277^T^	Eucalyptus, South Africa	MLFS00000000.1
*Pantoea* sp.	At-9b	Leaf cutter ant, USA	CP002433.1, CP002434.1, CP002435.1, CP002436.1, CP002437.1, CP002438.1
*Pantoea* sp.	A4	Rafflesia flower, Malaysia	ALXE00000000.1
*Pantoea* sp.	GM01	Poplar, USA	AKUI00000000.1
*Tatumella morbirosei*	LMG 23360^T^	Pineapple, Philippines	CM003276.1
*Tatumella ptyseos*	ATCC 33301^T^	Human, USA	ATMJ00000000.1
*Tatumella saanichensis*	NML 06-3099^T^	Human, Canada	ATMI00000000.1

### Generation of shared gene sets

The shared gene sets of the two genera, as well as the different lineages within *Pantoea* (see Figure 1), were generated with the EDGAR (Efficient Database framework for comparative Genome Analyses using BLAST score Ratios) server (https://edgar.computational.bio.uni-giessen.de; Blom et al., 2016). For each gene set, a representative of the lineage/genus was used for downstream analyses. The representatives used for the different lineages were *P. agglomerans* R190 for the first lineage (encompassing *P. agglomerans, P. eucalypti, P. vagans, P. deleyi, P. anthophila, P. brenneri*, and *P. conspicua*), *P. ananatis* LMG 2665^T^ for the second lineage (comprising of *P. ananatis, P. allii, P. stewartii* subsp. *stewartii*, and *P. stewartii* subsp. *indologenes*), *P. dispersa* strain number to EGD-AAK13 for the third lineage (encompassing *P. dispersa, P. eucrina*, and *P. wallisii*) and *P. rodasii* LMG 26273^T^ for the fourth lineage (consisting of *P. rodasii, P. rwandensis*, and *Pantoea* sp. GM01). For the intergeneric comparisons, *P. agglomerans* R190 was again used as representative of *Pantoea* and *T. ptyseos* ATCC 33301^T^ as representative of *Tatumella*.

### Functional annotation and identification of differentially present metabolic pathways

Functional annotation of the different gene sets were first performed by orthology searches against the KEGG database (Kanehisa et al., 2016b) using GhostKOALA (KEGG Orthology and Links Annotation; Kanehisa et al., 2016a) for all gene sets. Genes with KO terms associated with them could be separated based on the functional role of the pathways to which they could be mapped. Specific pathways where differences were detected in the global maps were also considered for comparative purposes (Figure 1).

For the *Pantoea* lineages, genes with no KO associations were then analyzed to assign putative functions using Blast2GO (Conesa et al., 2005; Götz et al., 2008). This was done by subjecting these genes to BLAST analyses for Gene Ontology (GO) associations using Blast2GO implemented in CLC Genomics Workbench (CLC Bio). In these analyses, assignment to more than one GO term per gene was allowed when functional annotation suggested that a gene product is involved in multiple processes. All Blast2GO analyses were initiated by BLAST searches against the RefSeq non-redundant protein database of NCBI followed by InterproScan (Jones et al., 2014) analyses to identify protein domains as a means of identifying putative functions. Genes remaining without annotation was again subjected to BLAST analyses against the non-redundant database on NCBI to determine the distribution of these genes across taxa.

Individual sets of reconstructed metabolic pathways obtained from the KEGG database were compared to identify differences between lineages and genera. This was done by assigning the set of KEGG pathway maps from each genus/lineage a unique color and then overlaying them onto each other for identifying differences (Figure 1). From these overview pathway maps, specific metabolic pathways were identified for further investigation in eight functional categories used in KEGG. These were carbohydrate, lipid, nucleotide, amino acid and energy metabolism, as well as genes involved in environmental information processing and the metabolism of co-factors, vitamins, and xenobiotics.

Multi-gene pathways that were differentially present or absent were identified from the full set of differences obtained from the KEGG pathway comparisons. This was done to limit the number of genes potentially identified as absent due to sequencing or assembly errors and also aided in simplifying the overall analysis. For this purpose multi-gene pathways were defined as processes where more than one gene was required to complete a process. From these pathways, the absence of genes from the genomes included in the respective gene sets were verified using local BLAST (Altschul et al., 1990) analyses (tblastn). The genomic coordinates of these genes were then noted to identify clustered genes. The gene clusters were identified and visualized using Geneious 6.1.6 (Biomatters).

Sequences for complete clusters were subsequently extracted from genomes and aligned using the MAFFT 7.309 (Katoh and Standley, 2013) server. When more than three members of a lineage possessed the gene clusters, their sequence alignments were subjected to codon-based selection analyses in MEGA 6.0.6 (Tamura et al., 2013) using HyPhy (Pond and Muse, 2005), to obtain dN (proportion of non-synonymous substitutions) and dS (proportion of synonymous substitutions) values at all codon positions across the alignments. The normalized dN-dS values were then plotted against codon positions in Microsoft Excel 2013.

## Results

### Generation of shared gene sets

**Figure 2:**
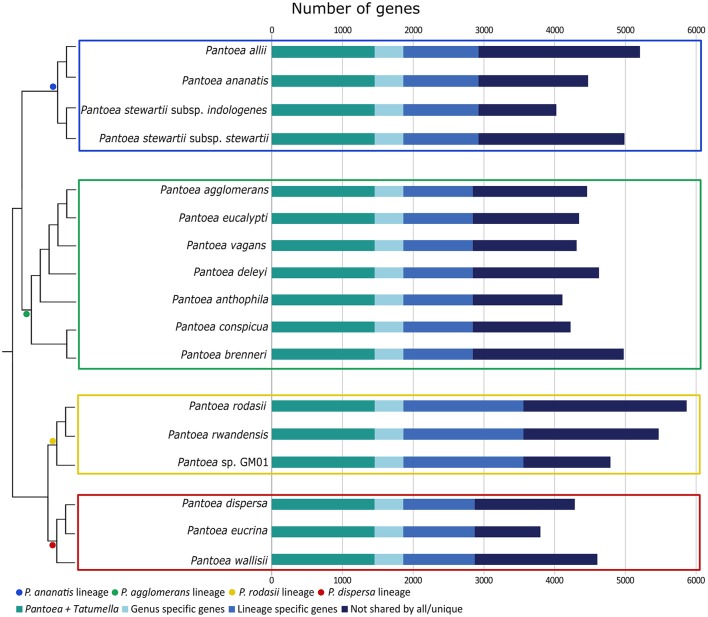
A bar graph indicating the number of genes for each isolate, separated into genes highly conserved in the sister genera (*Pantoea* + *Tatumella*), through to genes not shared by all closely related species or unique genes. The dendrogram was inferred for the different lineages and their relationships to each other from the amino acid based topology of the core genome of Palmer et al. (2017). The length of each bar is indicative of the size of each genome analyzed (in terms of the number of genes). The different lineages are indicated with colored blocks (blue—*P. ananatis* lineage; green—*P. agglomerans* lineage; yellow—*P. rodasii* lineage; red—*P. dispersa* lineage). All genomes analyzed encoded a similar number of genes, with the genome of *P. rodasii* encoding the highest number of genes (~5,800) and *P. eucrina* encoding the least number of genes (~3,800).

**Figure 3:**
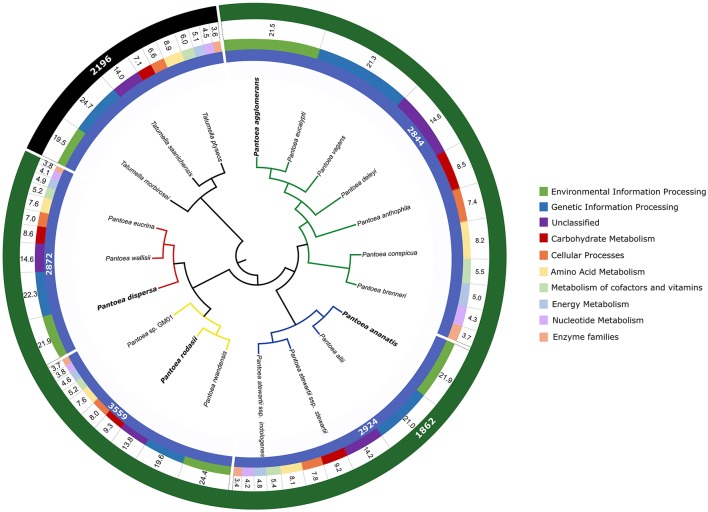
An overview of the gene sets shared between *Pantoea* and *Tatumella* and among the various lineages of *Pantoea*. For perspective, these are indicated relative to the genome-based species tree for *Pantoea* generated by Palmer et al. (2017). The lineages examined in this study are indicated with colored branches (green—*P. agglomerans* lineage; blue—*P. ananatis* lineage; yellow—*P. rodasii* lineage; red—*P. dispersa* lineage) with the representative of each lineage indicated in bold. The inner track indicates the sizes of the gene sets (in number of genes) of the lineages within *Pantoea*. The second track indicates the proportion of the genes annotated with the KEGG database involved in each of the functional classes (see legend). Values in the third track depicts the percentage of annotated genes involved in each functional class. The outer track indicates the size of the shared gene sets for the genera *Pantoea* and *Tatumella*, respectively.

In this study, we identified a number of biologically informative characters for *Pantoea* and the four lineages examined. For analyses at both the inter- and intra-generic levels, comparable taxon sets were compiled based on phylogenetic relatedness and Average Nucleotide Identity values (ANI Konstantinidis and Tiedje, 2005b; see Palmer et al., 2017). At the intra-generic level, these sets were also comparable in the sizes of the gene sets (Figure 2), with the exception of the *P. rodasii* lineage. This larger gene set could be attributed to the large size of the genomes of the three current members in the lineage, in comparison to the members of other lineages. However, a large proportion of the genes (>25%) in the respective genomes were present in both *Pantoea* and *Tatumella*. For the *Pantoea* comparisons more than 30% of the genes in respective genomes were present in all taxa, with 55–75% associated with specific lineages and 25–45% apparently species-specific. Overall, the gene sets for the four lineages consisted of 2844 genes for the *P. agglomerans* lineage, 2924 genes for the *P. ananatis* lineage, 3599 genes for the *P. rodasii* lineage and 2872 genes for the *P. dispersa* lineage (Figure 3).

For the inter-generic comparisons, the *Pantoea* gene set (calculated from 21 genomes) consisted of 1862 genes. The *Tatumella* gene set consisted of 2196 genes (calculated from three genomes). This difference in the number of shared genes can most likely be attributed to the number of genomes analyzed in these genera, as the number of genomes available for *Tatumella* is underrepresented.

### Functional annotation of the *Pantoea* and *Tatumella* gene sets with KEGG

The number of genes with KO associations for *Pantoea* and *Tatumella* were 1,576 (84.6% of the *Pantoea* gene set) and 1,760 (80.1% of the *Tatumella* gene set), respectively (Supplementary File S1). In both cases, the highest number of genes was involved in “Genetic Information Processing”, followed by “Environmental Information Processing”, with “Unclassified” genes making up the third largest gene group.

The pathways in which we identified differences between *Pantoea* and *Tatumella* were “Metabolic pathways”, “Biosynthesis of secondary metabolites”, “Microbial metabolism in diverse environments”, “Biosynthesis of antibiotics”, “Carbon metabolism”, “Biosynthesis of amino acids”, as well as “2-Oxocarboxylic acid metabolism”, and “Fatty acid metabolism”. Comparison of the relevant global metabolic maps revealed a higher number of reactions predicted for *Tatumella* (as would be expected due to the higher number of shared genes), except for “Fatty acid metabolism”. Closer inspection of the fatty acid metabolism pathways indicated the ability to perform β-oxidation of fatty acids occurred in *Pantoea* but not *Tatumella*.

A total of 124 differences were identified between *Pantoea* and *Tatumella* (Supplementary File S1). These consisted of reactions involved in all functional classes, namely “Carbohydrate metabolism” (citrate cycle, pentose phosphate pathway, fructose and mannose metabolism, ascorbate and aldarate metabolism, starch and sucrose metabolism, glyoxylate and dicarboxylate metabolism and inositol phosphate metabolism), “Energy metabolism” (including methane, sulfur and nitrogen metabolism), “Lipid metabolism” (including fatty acid degradation and sphingolipid metabolism), “Nucleotide metabolism” (purine and pyrimidine metabolism), “Amino acid metabolism” (cysteine and methionine metabolism, lysine degradation, arginine and proline metabolism, histidine metabolism and β-alanine metabolism), “Cofactor metabolism” (nicotinate and nicotinamide metabolism), “Xenobiotics metabolism” (benzoate degradation, chloroalkane and chloroalkene degradation) and “Environmental information processing” [ABC transporters, two-component systems, phosphotransferase systems (PTSs) and chemotaxis]. By limiting the pathways investigated to those where two or more genes are required to complete a pathway, 10 pathways (32 differences) were retained and subsequently absence was confirmed with BLAST analyses (Table 2, Supplementary File S1).

**Table 2:** Intergeneric differences in multi-gene pathways.

**Process^a^**	**Genes**	**Biological function**	**Distribution across lineages^b^**	**References**
D-ribose utilization	*rbsK, pgm*	Converts D-ribose to D-ribose-1-P Ribose donor during pyrimidine cycling Involved in co-factor cycling	*Pantoea* (21/21) *Tatumella* (0/3)	Giorgelli et al., 1997; Gossmann et al., 2012; Armenta-Medina et al., 2014
D-mannitol utilization	*mtlAD*	Converts D-mannitol to β-D-fructose-6-P Used as carbon source	*Pantoea* (21/21) *Tatumella* (0/3)	Berkowitz, 1971; Sprenger, 1993; Boer et al., 1996
Myo-inositol utilization	*iolBCDEG*	Converts 2-deoxy-5-keto-D-gluconate-6-P to myo-inositol Used as carbon source	*Pantoea* (21/21) *Tatumella* (0/3)	Anderson and Magasanik, 1971; Yoshida et al., 2004
Sucrose utilization	sacA, MGAM, SI	Degrades sucrose to ADP-glucose and D-fructose Used as carbon source	*Pantoea* (21/21) *Tatumella* (0/3)	Fouet et al., 1986; Van Beers et al., 1995; Sim et al., 2008
Glycogen degradation	*treXYZ, glgB*	Converts glycogen to trehalose or amylose Used as carbon source	*Pantoea* (21/21) *Tatumella* (0/3)	Baecker et al., 1986; Amemura et al., 1988; Chandra et al., 2011
β-oxidation	*fadABE*	Cleaves fatty acids cyclically into acetyl-CoA (even numbered) or succinyl-CoA (odd-numbered) Used in citric acid cycle	*Pantoea* (21/21) *Tatumella* (0/3)	Schulz, 1991; Fujita et al., 2007; Liu et al., 2010
Purine metabolism	*guaD, XDH, uaZ, pucM, PRHOXNB*	Converts guanine to (S)-allantoin Process involved in nitrogen cycling	*Pantoea* (21/21) *Tatumella* (0/3)	Kimiyoshi et al., 1993; Colloc'h et al., 1997; Nygaard et al., 2000; Moffatt and Ashihara, 2002; Cendron et al., 2007
Creatine degradation	E3.5.3.3, E3.5.1.59	Converts creatine and N-carbamoylsarcosine to sarcosine	*Pantoea* (21/21) *Tatumella* (0/3)	Yoshimoto et al., 1976; Deeg et al., 1987; Suzuki, 1991
Histidine degradation	*hisD, hutH*	Converts L-histidinol to urocanate Use of histidine as sole carbon source	*Pantoea* (21/21) *Tatumella* (0/3)	Mehler and Tabor, 1953; Schwede et al., 1999; Yang et al., 2011
L-aspartate degradation	*nadABCX*	Converts L-aspartate to nicotinate-D-ribonucleotide Process involved in co-factor cycling	*Pantoea* (21/21) *Tatumella* (0/3)	Yang et al., 2003; Ollagnier-de Choudens et al., 2005; Katoh et al., 2006; Gossmann et al., 2012
Glutamine ABC Transporter	*glnHPQ*	Transports glutamine from outside environment into cell through the use of ATP	*Pantoea* (0/21) *Tatumella* (3/3)	Nohno et al., 1986; Walshaw et al., 1997; Hosie and Poole, 2001
Glycine/ betaine/proline ABC Transporter	*proVWX*	Transports glycine, betaine and proline from outside environment into cell through the use of ATP	*Pantoea* (0/21) *Tatumella* (3/3)	Gowrishankar, 1989; Stirling et al., 1989; Kempf and Bremer, 1995; Ko and Smith, 1999
Glutathione ABC Transporter	*gsiABCD (yliABCD)*	Transports glutathione from outside environment into cell through the use of ATP	*Pantoea* (0/21) *Tatumella* (3/3)	Suzuki et al., 2005
Osmoprotectant ABC Transporter	*opuABCD*	Transports osmoprotectants from outside environment into cell through the use of ATP	*Pantoea* (21/21) *Tatumella* (0/3)	Kempf and Bremer, 1995; Kappes et al., 1999
Taurine ABC Transporter	*tauABC*	Transports taurine from outside environment into cell through the use of ATP Allows uptake for subsequent degradation of taurine for sulfur cycling	*Pantoea* (21/21) *Tatumella* (0/3)	Van der Ploeg et al., 1996; Javaux et al., 2007
L-arabinose transporter	*araFGH*	Transports L-arabinose from outside environment into cell through the use of ATP Required for utilization of L-arabinose as sole carbon source	*Pantoea* (21/21) *Tatumella* (0/3)	Scripture et al., 1987; Kehres and Hogg, 1992; Schneider, 2001
Microcin C ABC Transporter	*yejABEF*	Transports microcin C from outside environment into cell through the use of ATP Results in susceptibility to antimicrobial microcin C	*Pantoea* (21/21) *Tatumella* (0/3)	Vanneste et al., 2001; Novikova et al., 2007; Metlitskaya et al., 2009
N-acetylmuramic acid PTS	*murP, crr*	Transports and phosphorylates N-acetylmuramic acid through the use of ATP Essential for the use of N-acetylemuramic acid (cell wall component) as carbon source	*Pantoea* (21/21) *Tatumella* (0/3)	Dahl et al., 2004; Uehara et al., 2006,
N-acetyl-D-glucosamine PTS	*nagE*	Transports and phosphorylates N-acetyl-D-glucosamine through the use of ATP Essential for the use of N-acetyl-D-glucosamine (cell wall component) as carbon source	*Pantoea* (0/21) *Tatumella* (3/3)	Jaeger and Mayer, 2008; Plumbridge, 2009
Arbutin/salicin PTS	*ascF, crr*	Transports and phosphorylates arbutin and salicin through the use of ATP Essential for the use of plant derived glycosides as sole carbon source	*Pantoea* (0/21) *Tatumella* (3/3)	Hall and Xu, 1992; Dahl et al., 2004

a *PTS refers to phosphotransferase system*.

b *Brackets indicate the number of taxa for which the specific locus is present out of all taxa in the genus*.

As suggested from the global maps, genes required for β-oxidation of long-chain fatty acids (“Lipid metabolism”—fatty acid degradation) were present in all members of *Pantoea* and absent in all members of *Tatumella*. A number of genes involved in specific pathways in carbohydrate metabolism where detected only in *Pantoea*, namely the “Pentose phosphate pathway” (D-ribose to D-ribose-1-P), “Fructose and mannose metabolism” (D-mannitol to β-D-fructose-6-P), “Starch and sucrose metabolism” (sucrose to ADP-glucose; glycogen to trehalose and amylose, respectively) and “Inositol phosphate metabolism” (myo-inositol to 2-deoxy-5-keto-D-gluconate-6-P). The “Energy metabolism” pathway with differences was sulfur metabolism, where *Pantoea* possessed genes required for the uptake of extracellular taurine and its subsequent conversion to sulfite. *Pantoea* also possessed genes required for the conversion of guanine to (S)-allantoin during “Purine metabolism”. Several pathways involved in “Amino acid metabolism” were also present only in *Pantoea*, specifically those involved in arginine and proline metabolism (creatine and N-carbamoylsarcosine to sarcosine) and histidine metabolism (L-histidinol to urocanate). In addition, *Pantoea* also possessed genes for the conversion of L-aspartate to nicotinate-D-ribonucleotide during “Cofactor metabolism” (nicotinate and nicotinamide metabolism).

We also observed differences for genes in the category “Environmental Information Processing”. Amongst the ABC transporters, those for glutamine, glutathione and glycine betaine/proline transporters were found only in *Tatumella*, while those for osmoprotectant, taurine (also seen in sulfur metabolism), L-arabinose, and microcin C were only present in *Pantoea*. In terms of the phosphotransferase systems, *Pantoea* possessed genes necessary to transport and convert N-acetylmuramic acid to N-acetylmuramic acid-6-P, while *Tatumella* possessed genes required for the transport and conversion of N-acetyl-D-glucosamine to N-acetyl-D-glucosamine-6-P and arbutin/salicin to arbutin-6-P/salicin-6-P.

### Functional annotation of the genes shared by lineages in *Pantoea* with KEGG

For the *P. agglomerans* lineage, the number of shared genes with KO associations resulted in 2148 genes (75.5% of the gene set). A total of 2197 genes (75.1% of the gene set) in the *P. ananatis* lineage could be annotated using KO terms (Supplementary File S2). The highest percentage of genes with associated KO terms were 75.9% (2181 genes) for the *P. dispersa* lineage, with the lowest being 71.9% for the *P. rodasii* lineage (2559 genes) (Supplementary File S2). In contrast to the inter-generic gene sets, the highest number of genes in all four lineages were involved in “Environmental Information Processing”, followed by “Genetic Information Processing”, with “Unclassified” genes again being the third most prevalent (Figure 3, Supplementary File S2).

Comparisons of global maps indicated differences between the four lineages in “Biosynthesis of amino acids”, “Biosynthesis of antibiotics”, “Biosynthesis of secondary metabolites”, “Carbon metabolism”, “Overview metabolism” and “Microbial metabolism in diverse environments” (Supplementary File S2). Limiting the reactions investigated to two or more genes acting together to complete a pathway, led to the identification of a number of reactions involved in “Polyketide sugar unit biosynthesis”, “Biosynthesis of siderophore group non-ribosomal peptides”, “Starch and sucrose metabolism”, “Riboflavin metabolism”, “Fructose and mannose metabolism”, “Lysine degradation”, “Chloroalkane and chloroalkene degradation”, “Benzoate degradation”, “Pentose and glucuronate interconversions” and “Cysteine and methionine metabolism” (Supplementary File S3). However, we excluded “Starch and sucrose metabolism” and “Riboflavin biosynthesis” after local BLAST analyses showed that homologs of the respective genes were detected in all taxa (Supplementary File S3). They were likely not recognized previously in our generation of the shared gene datasets with EDGAR's strict orthology estimation criteria. The genes involved in the remaining nine processes (two of which were involved in siderophore synthesis) were all found to be clustered and allowed comparison of the gene clusters across all taxa containing these genes (Table 3).

**Table 3:** Distribution multi-gene pathways among the *Pantoea* lineages.

**Processes**	**Pathway reactions**	**Genes^a^**	***Pantoea*** **lineages**^**b**^			
			**Ag**	**An**	**Di**	**Ro**
Polyketide sugar unit biosynthesis	dTDP-4-oxo-6-deoxy-D-glucose <-> dTDP-4-dehydro-beta-L-rhamnose	*rfbC*	v	v	v	v
	dTDP-L-rhamnose <-> dDTP-4-dehydro-beta-L-rhamnose	*rfbD*	v	v	v	v
Biosynthesis of siderophore group non-ribosomal peptides	2,3-Dihydroxybenzoate <-> Enterochelin	*entE* & *F*	v	−	v	+
	2,3-Dihydroxybenzoate <-> (2S,3S)-2,3-Dihydro-2,3-dihydroxybenzoate	*entA*	v	−	v	+
	(2S,3S)-2,3-Dihydro-2,3-dihydroxybenzoate <-> Isochorismate	*entB*	v	−	v	+
	Isochorismate <-> Chorismate	*entC*	v	−	v	+
Fructose and mannose metabolism	L-Rhamnulose <-> L-Rhamnose	*rhaA*	+	v	v	+
	L-Rhamnulose 1-P <-> L-Rhamnulose	*rhaB*	+	v	v	+
	Glycerone-P + S-Lactaldehyde <-> L-Rhamnulose 1-P	*rhaD*	+	v	v	+
Lysine degradation	L-Lysine <-> N6-Hydroxy-L-Lysine	*iucD*	−	+	−	−
	N6-Hydroxy-L-lysine <-> N6-Acetyl-N6-hydroxy-L-lysine	*iucB*	−	+	−	−
	N6-Acetyl-N6-hydroxy-L-lysine <-> N2-Citryl-N6-acetyl-N6-hydroxy-L-lysine	*iucA*	−	+	−	−
	N2-Citryl-N6-acetyl-N6-hydroxy-L-lysine + N6-Acetyl-N6-hydroxy-L-lysine <-> Aerobactin	*iucC*	−	+	−	−
Chloroalkane degradation	Chloroacetic acid <-> Glycolate + Hydrochloric acid	2-haloacid DH	+	+	−	+
	Chloroacetaldehyde <-> Chloroacetic acid	aldehyde DH	+	+	−	+
Benzoate degradation	Succinyl-CoA + 3-Oxoadipate <-> Succinate + 3-Oxoadipyl-CoA	*pcaI* & *J*	−	+	−	−
	2-Oxo-2,3-dihydrofuran-5-acetate + H2O <-> 3-Oxoadipate	*pcaL*	−	+	−	−
	3,4-Dihydroxybenzoate + Oxygen <-> 3-Carboxy-cis,cis-muconate	*pcaG* & *H*	−	+	−	−
Pentose and glucuronate interconVersions	D-Glucuronate <-> D-fructuronate	*uxaC*	+	+	v	+
	D-Galacturonate <-> D-Tagaturonate	*uxaC*	+	+	v	+
	D-Altronate <-> D-Tagaturonate	*uxaB*	+	+	v	+
	D-Altronate <-> 2-Dehydro-3-deoxy-D-gluconate	*uxaA*	+	+	v	+

a *The presence or absence of each pathway was verified using BLAST searches with the relevant sequences against the respective genomes*.

b *The lineages are indicated as follows: Ag = P. agglomerans lineage, An = P. ananatis lineage, Di = P. dispersa lineage and Ro = P. rodasii lineage. The presence of genes are indicated with “+”, their absence with “−”, while “v” is used to indicate the presence in some but not all members of a lineage*.

**Figure 4:**
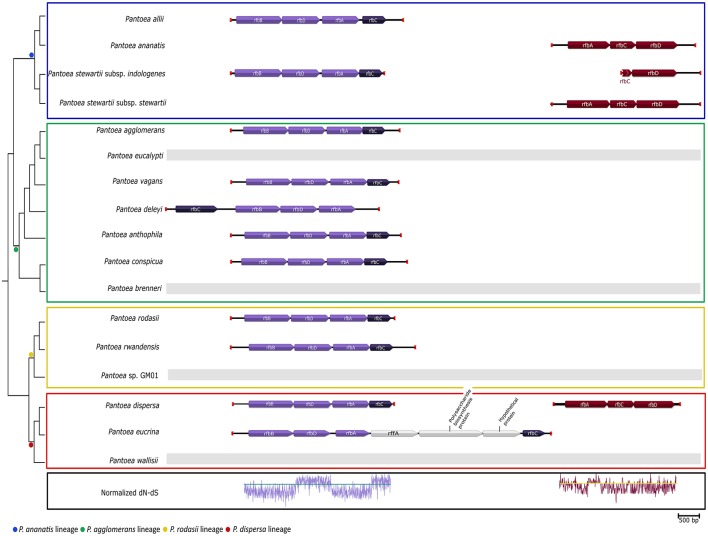
The gene cluster involved in “Polyketide sugar unit biosynthesis” across the lineages of *Pantoea*. The dendrogram was inferred from the amino acid based topology of the core genome of Palmer et al. (2017). The different lineages are indicated with colored blocks. The first locus, containing *rfbA, rfbB, rfbC*, and *rfbD* (purple), was conserved in most members of *Pantoea*. The normalized dN-dS values for each codon position were plotted as an indication of the selective pressures upon the codons. Both *rfbA* and *rbfB* could be observed to experience mainly purifying selection (proportion of non-synonymous substitutions < proportion of synonymous substitutions), while *rfbC* and *rfbD* evolved mainly under diversifying selection (proportion of non-synonymous substitutions > proportion of synonymous substitutions). The second locus was identified in *P. ananatis, P. stewartii* subsp. *stewartii* and *P. dispersa*, with a partial locus present in *P. stewartii* subsp. *indologenes* (maroon). This locus lacked an *rfbB* gene and evolved mainly under purifying selection.

The two genes identified (*rfbC* and *rfbD*) being involved in “Polyketide sugar unit biosynthesis” were present in all the members of the *P. ananatis* lineage, with various members of the other three lineages lacking the genes (*P. eucalypti, P. brenneri, Pantoea* sp. GM01 and *P. wallisii*; Table 3). Upon examination of the gene cluster containing these genes, two different loci were identified (Figure 4). The first locus was observed in nearly all members of the genus that possessed these genes (including *P. dispersa* and *P. stewartii* subsp. *indologenes*), while the second locus (lacking *rfbB*) was found only in *P. ananatis, P. stewartii* subsp. *stewartii, P. dispersa* and a partial locus in *P. stewartii* subsp. *indologenes*. The first locus was also slightly different in *P. deleyi* and *P. eucrina*, as the position of *rfbC* in *P. deleyi* differed (Figure 4—gene indicated in a darker shade) and the locus of *P. eucrina* contained an additional three genes in comparison to the other taxa (Figure 4). Furthermore, selection analyses indicated purifying selection for *rfbA* and *rfbB* and diversifying selection for *rfbC* and *rfbD* in the first locus (Figure 4, Supplementary File S4). Contrary to this, both *rfbC* and *rfbD* were under purifying selection in the second locus, with *rfbA* being under mainly purifying selection for the first part and diversifying selection for the second part of the gene (Figure 4, Supplementary File S4).

**Figure 5:**
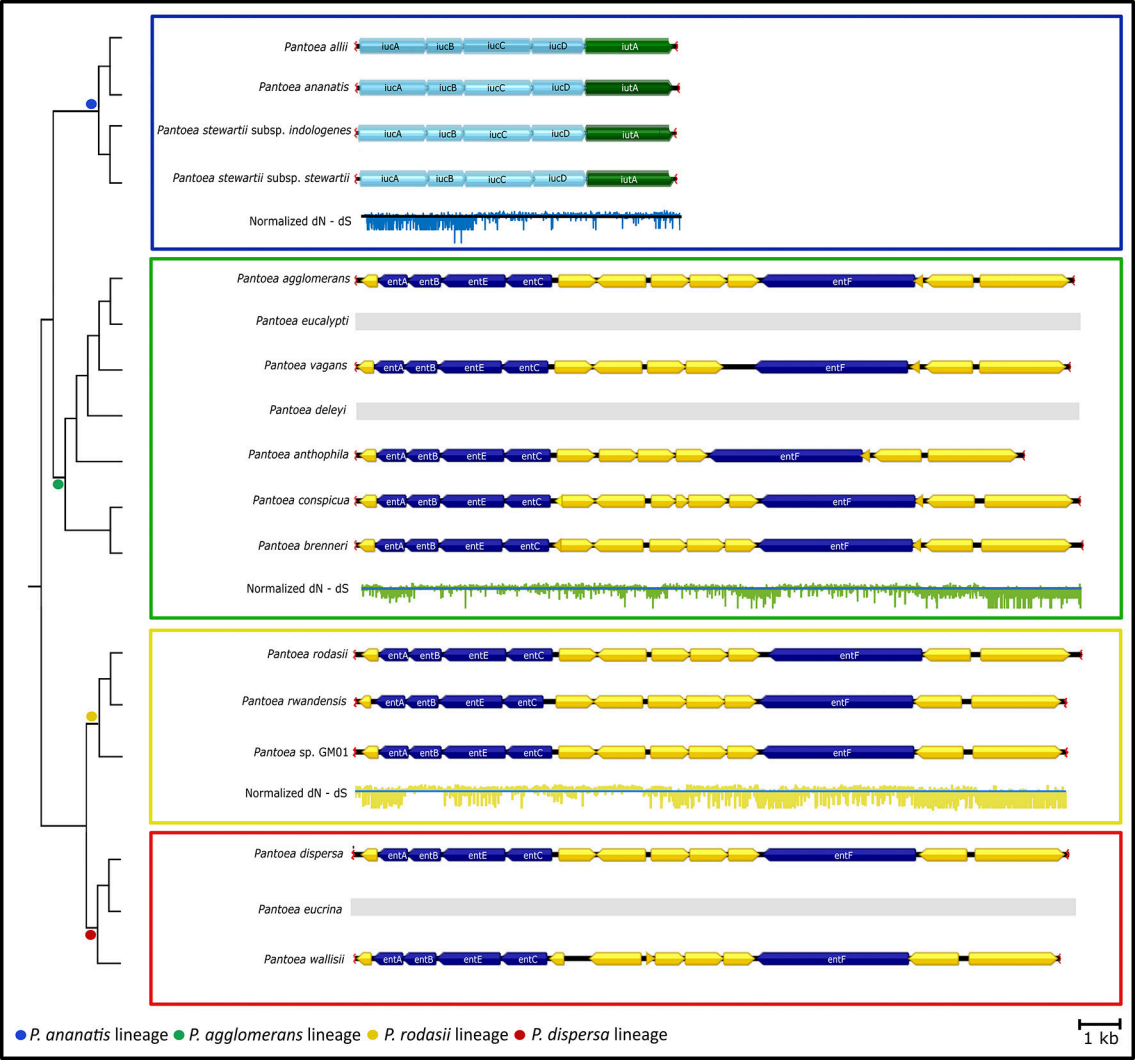
The gene clusters involved in “Lysine degradation” and “Biosynthesis of siderophore group non-ribosomal peptides”. The dendrogram was inferred from the species tree of Palmer et al. (2017). Lineages are indicated with colored blocks. Both these clusters encode for the biosynthesis of siderophores, namely aerobactin and enterobactin, respectively. The locus required for the production of aerobactin was conserved in members of the *P. ananatis* lineage, while the locus required for enterobactin biosynthesis was present in most other members of *Pantoea*. The enterobactin biosynthesis locus was completely absent from the genomes of the members of the *P. ananatis* lineage, while the aerobactin locus was lacking in all other members of *Pantoea*. As an indication of selective pressures on the loci, the normalized dN-dS value at each codon position was plotted across the clusters.

The genes involved in “Biosynthesis of siderophore group non-ribosomal peptides” and “Lysine degradation” both encoded for different iron acquisitioning molecules (siderophores) (Table 3). The genes present in most of the members in the genus (“Biosynthesis of siderophore group non-ribosomal peptides”) were identified as being required for the production of enterobactin. The majority of the gene cluster encoding enterobactin appeared to have evolved under purifying selection, with only some regions that evolved mainly under diversifying selection (for example see Figure 5 *entB*; Supplementary File S4). Conversely, the genes involved in “Lysine degradation” in the *P. ananatis* lineage were those required to produce aerobactin from lysine. All members of the *P. ananatis* lineage lacked the genes involved in enterobactin biosynthesis, but contained the genes required for aerobactin synthesis, while all other members of the genus lacked the genes required for aerobactin biosynthesis (Figure 5). Selection analyses amongst the genes encoding for aerobactin biosynthesis indicated purifying selection in particular for *iucA* and *iucB*. Both these loci were absent from *P. eucalypti, P. deleyi* and *P. eucrina*.

The differentially present genes associated with “Fructose and mannose metabolism” consisted of *rhaA, rhaB*, and *rhaD* which convert L-rhamnose to glycerone-P and S-lactaldehyde (*rhaD*) (Table 3). This cluster was present in all the members of the *P. agglomerans* and *P. rodasii* lineages, but present only in *P. allii* and *P. ananatis* in the *P. ananatis* lineage, and *P. dispersa* and *P. wallisii* in the *P. dispersa* lineage (Supplementary File S4). From the selection analyses of the *P. agglomerans* and *P. rodasii* lineages it was observed that *rhaB* and *rhaD* evolved under purifying selection, with *rhaA* evolving under diversifying selection (Supplementary File S4).

Our analysis showed that for “Chloroalkane and chloroalkene degradation”, the specific pathway was absent from all the members of the *P. dispersa* lineage (Table 3). This pathway catalyzes the conversion of chloroacetaldehyde to glycolate and hydrochloric acid. All other members of the genus possessed the genes required for this process (Supplementary File S4).

The differentially present genes associated with “Benzoate degradation” were involved in the utilization of protochatechuate. They were present only in the *P. ananatis* lineage. However, closer examination revealed that most members of the lineage contained a cluster of 9 genes (*pcaH, pcaG, pcaQ, pcaL, pcaB*, KAT, *pcaJ, pcaI*, and *pcaR*), but that it contained a deletion in *P. stewartii* subsp. *stewartii* which truncated *pcaL* and removed *pcaB* and KAT from the cluster. Overall, the cluster appeared to be under purifying selection (Supplementary File S4).

All members of the genus, except *P. eucrina*, possessed a gene cluster (*uxaA, uxaB*, and *uxaC*) involved in “Pentose and glucuronate interconversions” (Table 3, Supplementary File S4). The products of *uxaA* and *uxaB* catalyze, respectively, the reversible conversion of 2-dehydro-3-deoxy-D-gluconate to D-altronate and D-altronate to D-tagaturonate, while *uxaC* facilitates interconversions between D-tagaturonate and D-glucuronate and between D-fructuronate and D-galacturonate. These three genes were conserved within the *P. agglomerans, P. ananatis*, and *P. rodasii* lineages, with only *uxaA* and *uxaB* being present in *P. dispersa* and *P. wallisii*. Overall, it appeared that these genes were evolving under neutral selection (Supplementary File S4).

Comparison of the processes involved in “Cysteine and methionine metabolism” showed differences in the synthesis of spermidine (*speD* and *speE*) and the methionine salvage pathway (*mtnA, mtnB, mtnC, mtnD*, and *mtnK*) (Table 3, Supplementary Files S3, S4). The two genes required for the biosynthesis of spermidine allow for the conversion of S-adenosyl-L-methionine and putrescine to 5′-methylthioadenosine and spermidine (*speE*). These two genes were present in all members of the genus except *Pantoea* sp. GM01 (*P. rodasii* lineage), *P. eucrina* and *P. wallisii* (both from the *P. dispersa* lineage; Supplementary File S4). Genes involved in the methionine salvage pathway allow conversion of 5-methylthio-D-ribose to 3-(methylthio)-propanoate through 5 different intermediate reactions (Supplementary File S3). These methionine salvage pathway genes were absent in the *P. dispersa* lineage (*P. dispersa, P. eucrina*, and *P. wallisii*) (Supplementary File S4).

**Figure 6:**
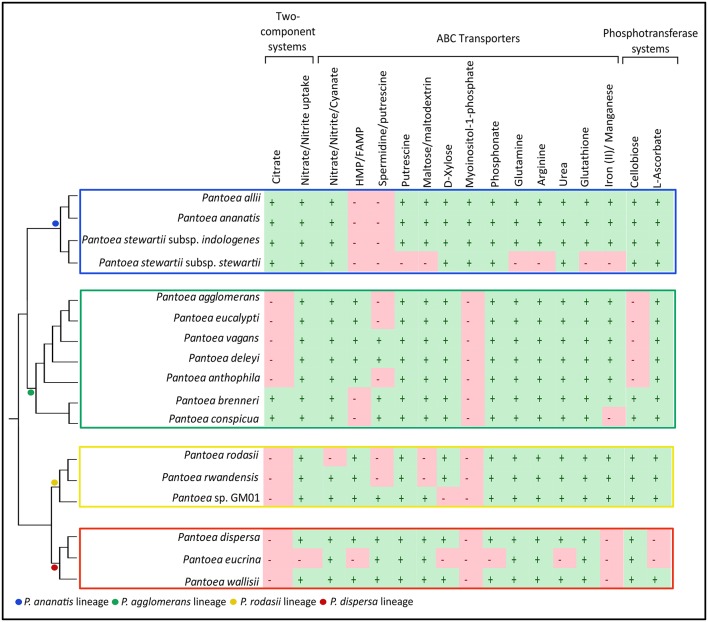
Differences between the lineages in processes involved in “Environmental Information Processing”. The presence (+) or absence (−) of complete (all genes required for functional system) two component systems, ABC transporters and PTSs in the genomes of the species in the main lineages within *Pantoea*. The dendrogram of the relationships within and between lineages were inferred from Palmer et al. (2017). The separate lineages are indicated with colored blocks.

We also found several multi-gene systems for two-component systems (2 systems), ABC Transporters (14 systems) and PTSs (2 systems) that were differentially present within these lineages (Supplementary File S5). Local BLAST analyses allowed identification of taxa where these genes were indeed present, despite not being conserved within the specific gene sets (Figure 6, Supplementary File S5). The two-component systems identified were that for citrate as well as nitrate/nitrite uptake. The ABC transporters identified were the systems for nitrate/nitrite/cyanate, HMP/FAMP, spermidine/putrescine, putrescine, maltose/maltodextrin, D-xylose, myoinositol-1-phophate, phosphonate, glutamine, arginine, urea, glutathione and iron(II)/manganese. The PTSs detected were those for cellobiose and L-ascorbate.

### Annotation of lineage-specific genes without KEGG associations

A total of 264 genes were identified as being differentially present in the *Pantoea* lineages, to which no KO term assignment could be made. This set of uncharacterized genes consisted of 62 genes in the *P. ananatis* lineage, 75 genes in the *P. agglomerans* lineage, 98 genes in the *P. rodasii* lineage and 29 genes in the *P. dispersa* lineage. Analysis of these genes with Blast2GO allowed annotation of 182 genes. A further six genes could be assigned GO terms, but could not be fully annotated upon merging of annotations due to a lack in InterProScan hits. A total of 76 genes had no functional associations. These unannotated genes could however be used for blastp analyses to identify potential sources of horizontally acquired genes.

**Figure 7:**
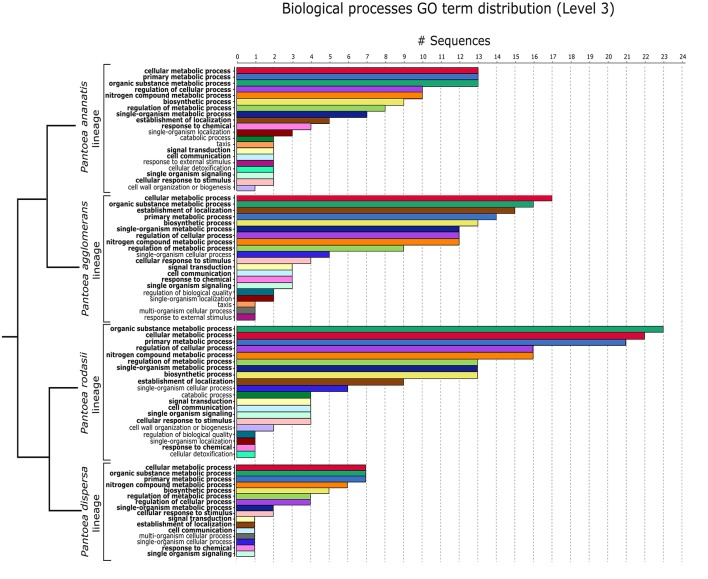
The top 20 biological processes (Level 3) for the *P. ananatis, P. agglomerans* and *P. rodasii* lineages and the 16 biological processes for the *P. dispersa* lineage, of the 182 genes annotated with Blast2GO. The dendrogram indicating the relationships between the lineages was inferred from the species tree of Palmer et al. (2017). The GO terms for the shared processes are indicated in the same color across the lineages. Processes highlighted in bold are shared by all four lineages.

The 62 genes of the *P. ananatis* lineage were subjected to Blast2GO analyses, leading to the annotation of 38 genes. In terms of biological processes (GO Level 3), the highest number of genes were involved in “cellular metabolic processes”, “primary metabolic processes” and “organic substance metabolic processes”, followed by “regulation of cellular processes” and “nitrogen compound metabolic processes” (Figure 7). This lineage thus contained 24 genes present in all members of the lineage, without KEGG or GO functional annotations. Based on blastp hits, 15 of the 24 genes had their closest homologs within other members of the *Enterobacteriaceae*, while two genes had homologs in members of the *Rhizobiaceae* (α-Proteobacteria). The closest homolog for three genes was respectively from the *Aurantimonadaceae* (*Martelella mediterranea*; α-Proteobacteria), *Corchorus olitorius* (bush okra; Malvaceae, Eudicots), and *Erwinia* phage ENT90. The remaining four genes had no BLAST hits (blastp) on the non-redundant database (Supplementary File S6).

Of the 75 genes conserved within the *P. agglomerans* lineage not annotated with KEGG, 54 genes could be annotated with Blast2GO. Most of these genes were involved, in descending order, in “cellular metabolic processes”, “organic substance metabolic processes”, “establishment of localization”, “primary metabolic processes” and “biosynthetic processes” (BP GO Level 3; Figure 7). The remaining 21 genes with no associated GO terms could not be functionally classified. Homologs of these genes were however, identified in other members of the *Enterobacteriaceae*, often pathogens, with a single gene having its closest homolog in the metagenome of a soil sample from an unknown source (Supplementary File S6).

Of the 98 genes conserved within the *P. rodasii* lineage without KEGG annotations, 76 could be annotated with Blast2GO. The five highest biological processes in which these genes were involved were “organic substance metabolic processes”, “cellular metabolic processes”, “primary metabolic processes”, “regulation of cellular processes” and “nitrogen compound metabolic processes” (GO Level 3; Figure 7). This resulted in 22 genes without any functional annotation with either KEGG or GO analyses. Homologs for all 22 genes were identified in other members of the *Enterobacteriaceae*, of which 21 genes were most closely related to genes from human pathogens (Supplementary File S6).

Of the 29 unique genes in the *P. dispersa* lineage without any KEGG annotations, 14 genes could be annotated with Blast2GO. These genes were primarily involved in “cellular metabolic processes”, “organic substance metabolic processes”, “primary metabolic processes”, “nitrogen compound metabolic processes” and “biosynthetic processes” (Figure 7). Of the 15 unannotated genes, homologs for all genes were identified in other members of the *Enterobacteriaceae*, particularly those associated with the stinkbug, *Plautia stali* (Supplementary File S6).

## Discussion

Our findings suggest that *in silico* mining of bacterial genome sequences is a feasible approach for inferring large sets of biological characters for particular taxa. This approach is invaluable for unveiling large repertoires of potential bacterial phenotypes and can thus contribute hugely toward identifying biologically relevant diagnostic characteristics from whole genome sequences. Furthermore, by superimposing such characters onto the phylogeny of a particular bacterial group it appears to be possible to identify those traits that might have contributed toward the initial emergence of a taxon and/or its subsequent stable persistence in nature. Here we identified extensive sets of biological characters specific to *Pantoea* and its main phylogenetic lineages. Our study thus outlines the initial steps toward linking biological functions (based on the variable genomic components) to taxonomy (based on the stable, conserved genomic components).

### Genome-based comparisons of specific processes between genera

The methodology employed in this study allowed for the identification of biological characters that potentially define and differentiate bacterial genera from one another. Despite the necessity of these taxonomic ranks, our understanding of what constitutes and distinguishes genera is mostly limited. Previous attempts to obtain natural and logical groupings have always been based on a limited view of the organisms' metabolic potential, often with a focus on what was considered to be clinically relevant data rather than from a biological outlook (Konstantinidis and Tiedje, 2005a,b; Sutcliffe, 2015). Although, the current classification system aims to identify and describe naturally occurring groups by employing an evolution-based approach, it still does not provide any biologically meaningful information for the organisms (Cohan, 2002; Konstantinidis and Tiedje, 2005a; Tindall et al., 2010). However, our study of *Pantoea* and *Tatumella* clearly highlights how diverse sets of biological characters for bacterial genera may be inferred from genome data. Apart from so-called genus-defining traits that can potentially be used to differentiate these taxa, these characters also provide information on the general biology of the taxa investigated (see Table 3). Our findings indicate that such genome-based analyses provide a more informed view of the biology of the organisms, and the information emerging from comparing metabolic differences can be linked to the shared ancestry of groups of organisms.

*Pantoea* appears to be metabolically more versatile than its sister genus *Tatumella*. Different from *Tatumella*, it encodes a range of additional pathways potentially enabling it to use diverse compounds as nutrient sources [e.g., fatty acids (Schulz, 1991; Fujita et al., 2007; Liu et al., 2010) and various carbohydrate derivatives (Mehler and Tabor, 1953; Anderson and Magasanik, 1971; Berkowitz, 1971; Yoshimoto et al., 1976; Baecker et al., 1986; Fouet et al., 1986; Deeg et al., 1987; Amemura et al., 1988; Sprenger, 1993; Van Beers et al., 1995; Boer et al., 1996; Schwede et al., 1999; Yoshida et al., 2004; Sim et al., 2008; Chandra et al., 2011; Yang et al., 2011)]. *Pantoea* also encodes additional nutrient cycling or salvage systems [e.g., purine, pyrimidine and co-factor cycling (Kimiyoshi et al., 1993; Colloc'h et al., 1997; Giorgelli et al., 1997; Nygaard et al., 2000; Moffatt and Ashihara, 2002; Yang et al., 2003; Ollagnier-de Choudens et al., 2005; Katoh et al., 2006; Cendron et al., 2007; Gossmann et al., 2012; Armenta-Medina et al., 2014)]. These systems have been shown to allow for the recycling of compounds that are no longer utilized in the cell, and might enhance *Pantoea*'s ability to perform basic, yet essential cellular functions under nutrient limiting conditions (Krismer et al., 2014; Shimizu, 2014).

*Pantoea* and *Tatumella* differ markedly in terms of KEGG's “Environmental Information Processing” functional category, which includes all signaling and membrane transport pathways (Kanehisa et al., 2002). Various ABC transporters (Nohno et al., 1986; Scripture et al., 1987; Gowrishankar, 1989; Stirling et al., 1989; Kehres and Hogg, 1992; Kempf and Bremer, 1995; Van der Ploeg et al., 1996; Walshaw et al., 1997; Kappes et al., 1999; Ko and Smith, 1999; Hosie and Poole, 2001; Schneider, 2001; Vanneste et al., 2001; Suzuki et al., 2005; Javaux et al., 2007; Novikova et al., 2007; Metlitskaya et al., 2009) and phosphotransferase systems (PTSs) (Hall and Xu, 1992; Dahl et al., 2004; Uehara et al., 2006; Jaeger and Mayer, 2008; Plumbridge, 2009) were differentially present in the two genera. Among the various ABC transporters identified only in *Pantoea*, one has been associated with susceptibility to microcin C in the absence of a microcin C-specific efflux pump (Metlitskaya et al., 2009), which is part of a group of antibiotic produced by certain *Enterobacteriaceae* (Vanneste et al., 2001; Metlitskaya et al., 2009). The absence of this ABC transporter in *Tatumella* and the concomitant antibiotic resistance may increase ecological competitiveness of species exposed to these compounds (Hacker and Carniel, 2001). Ecological advantages are likely also obtained from some of the predicted PTSs, which have previously been linked to enhanced recycling of cell wall components under nutrient-poor conditions (e.g., PTSs involving N-acetylmuramic acid and N-acetylglucosamine; Jaeger and Mayer, 2008), and the uptake of plant-derived carbon compounds (e.g., PTS involving arbutin and salicin; Zangoui et al., 2015).

Taken together, these findings suggest that evolution has equipped *Pantoea* with extensive repertoires of metabolic processes that make them generally more versatile in their ability to adapt to changing environments. Compared to *Tatumella*, they can utilize a wider range of carbon sources and use available resources more efficiently by recycling metabolic byproducts. This potentially also provides them with a competitive advantage in nutrient-poor environments such as mammalian blood. The various genus-defining traits we identified for *Pantoea* may thus contribute to our understanding of the complex, and often opportunistic, relationships these species have with their plant and animal hosts (De Baere et al., 2004; Cruz et al., 2007; De Maayer et al., 2012a).

### Genome-based comparisons of specific processes between lineages of *Pantoea*

Comparisons of the metabolic processes inferred from whole genome sequences allowed for the identification of various sets of traits specific to one or more lineages of *Pantoea*. Based on previous work in diverse bacteria (including *Pantoea*), we attempted to correlate these processes to the lifestyles of the taxa investigated. Although a number of the identified processes were likely related to pathogenicity (see Table 4), most probably play roles in niche adaptation and utilization in a non-pathogenic capacity (see Table 5).

**Table 4:** Pathogenicity-associated processes with differences between the lineages.

**Process**	**Gene cluster**	**Function, biological role and contribution to pathogenicity**	**Distribution across lineages^a^**	**References**
O-antigen biosynthesis	*rfb* locus 1	Encodes for the synthesis of polysaccharide unit of O-antigen Cause variation on outer membrane of cells Assists in host immune response evasion	*P. ananatis* lineage (2/4) *P. agglomerans* lineage (5/7) *P. rodasii* lineage (2/3) *P. dispersa* lineage (2/3)	Stevenson et al., 1994; Whitfield, 1995; Wang and Reeves, 1998; Kohchi et al., 2006; Greenfield and Whitfield, 2012
	*rfb* locus 2	Encodes for the synthesis of the O-antigen's polysaccharide Cause variation on outer membrane of cells Assists in host immune response evasion	*P. ananatis* lineage (3/4) *P. dispersa* lineage (1/4)	Stevenson et al., 1994; Whitfield, 1995; Wang and Reeves, 1998; Kohchi et al., 2006; Greenfield and Whitfield, 2012
Siderophore production	*iuc* locus	Encodes for the synthesis of aerobactin Efficient at scanvenging iron during nutrient limitation Assists in resistance against iron-dependent antimicrobials	*P. ananatis* lineage (4/4)	Montgomerie et al., 1984; Williams and Carbonetti, 1986; Opal et al., 1990; Fecteau et al., 2001; Torres et al., 2001; Garcia et al., 2011; Gao et al., 2012
	*ent* locus	Encodes for the synthesis of enterobactin High affinity for iron, but susceptible to inactivation by lipocalin-2 produced by mammalian hosts Iron acquisitioning in hosts	*P. agglomerans* lineage (5/7) *P. rodasii* lineage (3/3) *P. dispersa* lineage (2/3)	Fiedler et al., 2001; Torres et al., 2001; Hubertus et al., 2003; Raymond et al., 2003; Garcia et al., 2011
Polyamine biosynthesis	*spe* locus	Synthesis of spermidine from S-adenosyl-L-methionine Involved in protein and nucleic acid biosynthesis, expression regulation, membrane functioning and ROS scavenging Contributes toward pathogenesis through involvement in biofilm formation, escape from phagolysosomes and toxin production	*P. ananatis* lineage (4/4) *P. agglomerans* lineage (7/7) *P. rodasii* lineage (2/3) *P. dispersa* lineage (1/3)	Khan et al., 1992; Ha et al., 1998; Gugliucci and Menini, 2003; Shah and Swiatlo, 2008; Pegg, 2016

a *Brackets indicate the number of taxa for which the specific locus is present out of all taxa in a lineage. For complete distribution patterns see Supplementary File S4*.

**Table 5:** Niche-associated processes (non-pathogenic) with differences between the lineages.

**Process**	**Gene cluster**	**Function, biological role and contribution to niche adaptation**	**Distribution across lineages^a^**	**References**
L-rhamnose utlization	*rha* locus	Convert L-rhamnose to S-lactaldehyde Catabolism of L-rhamnose as carbon source Potentially contributes toward niche expansion	*P. ananatis* lineage (2/4) *P. agglomerans* lineage (7/7) *P. rodasii* lineage (3/3) *P. dispersa* lineage (2/3)	Badía et al., 1985; Moralejo et al., 1993; Saxena et al., 2010
D-galacturonate utilization	*uxa* locus	Involved in the utilization of glucuronate and galacturonate as carbon sources Integral parts of plant cell walls Can contribute toward colonization of plants	*P. ananatis* lineage (4/4) *P. agglomerans* lineage (7/7) *P. rodasii* lineage (3/3) *P. dispersa* lineage (2/3 partial)	Walton, 1994; Hématy et al., 2009; Richard and Hilditch, 2009
Benzoate degradation	*pca* locus	Involved in the utilization of protochatechuate Catabolism of protochatechuate as carbon source Potentially contributes toward niche expansion	*P. ananatis* lineage (4/4) (note, *P. stewartii* ssp. *stewartii* contains deletion)	Song, 2009; Brady et al., 2011; Gueule et al., 2015
Chloroalkane and chloroalkene degradation	-	Conversion of chloroacetaldehyde to glycolate Chloroacetaldehyde binds to DNA and causes conformational changes leading to mutations Removes mutagen from environment and utilize it as carbon source via glycolate	*P. ananatis* lineage (4/4 *P. agglomerans* lineage (7/7) *P. rodasii* lineage (3/3)	Young Kim et al., 2007; Maciejewska et al., 2010
Methionine salvage pathway	*mtn* locus	Salvage methionine through the conversion of 5-methylthio-D-ribose to 3-(methylthio)-propanoate Allows sulfur cycling Can survive in low-sulfur environments	*P. ananatis* lineage (4/4 *P. agglomerans* lineage (7/7) *P. rodasii* lineage (3/3)	Sekowska et al., 2000, 2004; Albers, 2009

a *Brackets indicate the number of taxa for which the specific locus is present out of all taxa in a lineage. For complete distribution patterns see Supplementary File S4*.

The processes likely associated with pathogenesis, particularly in the *Enterobacteriaceae*, were those involved in O-antigen (Stevenson et al., 1994; Whitfield, 1995; Wang and Reeves, 1998; Kohchi et al., 2006; Greenfield and Whitfield, 2012), siderophore (Montgomerie et al., 1984; Williams and Carbonetti, 1986; Opal et al., 1990; Fecteau et al., 2001; Fiedler et al., 2001; Torres et al., 2001; Hubertus et al., 2003; Raymond et al., 2003; Garcia et al., 2011; Gao et al., 2012) and polyamine (Khan et al., 1992; Ha et al., 1998; Gugliucci and Menini, 2003; Shah and Swiatlo, 2008; Pegg, 2016) biosynthesis. Differences in the locus involved in O-antigen biosynthesis (of which some *Pantoea* species have two) were previously associated with a pathogen's ability to escape host responses (Whitfield, 1995; Kohchi et al., 2006; Greenfield and Whitfield, 2012). Our results further showed that all species in the *P. ananatis* lineage likely produce aerobactin, while many of those in the other lineages produce enterobactin. Although these siderophores essentially perform the same function, aerobactin is more efficient at scavenging iron during nutrient limitation, and may in some instances even assist in resistance against iron-dependent antimicrobials (Williams and Carbonetti, 1986; Torres et al., 2001; Garcia et al., 2011). Also, all of the examined species in the *P. agglomerans* and *P. ananatis* lineages are predicted to be capable of producing the polyamine spermidine (this process was detected in only some of the species in the other two lineages). Apart from their essential cellular functions (Ha et al., 1998; Shah and Swiatlo, 2008; Pegg, 2016), polyamines have been implicated in biofilm formation, escape from host phagolysosomes and toxin production and activity (Shah and Swiatlo, 2008).

The group of processes likely associated with niche adaptation and utilization were those related to nutrient metabolism and “Environmental Information Processing” (Supplementary Table S1). For example, other than those in the *P. dispersa* lineage, all *Pantoea* species were predicted to be capable of converting the environmental mutagen chloroacetaldehyde to glycolate, thus providing the dual means of disposing of the mutagen and accessing glycolate as carbon source (Young Kim et al., 2007; Maciejewska et al., 2010). Similarly, in all species, bar those of the *P. dispersa* lineage, the methionine salvage pathway likely allow increased efficacy under sulfur cycling in nutrient-poor conditions (Sekowska et al., 2000, 2004; Albers, 2009). Species in the *P. ananatis* lineage encode the benzoate degradation pathways, which likely enable their utilization of protocatechuate as carbon source (Song, 2009; Brady et al., 2011; Gueule et al., 2015). Additionally, differences were also observed in rhamnose (Badía et al., 1985; Moralejo et al., 1993; Saxena et al., 2010) and galacturonate (Walton, 1994; Hématy et al., 2009; Richard and Hilditch, 2009) utilization. The four lineages further differed in terms of their ability to transport various nutrients (e.g., the myoinositol-1-phosphate ABC transporter occurred only in the *P. ananatis* lineage). The same was also true for the predicted two-component signaling systems and other PTSs (e.g., except for two *P. agglomerans* lineage species, only the *P. ananatis* lineage encoded a two-component signaling system for citrate utilization) (Supplementary Table S1).

Overall, we could correlate the versatility in lifestyle and host to increased metabolic potential (in terms of compounds utilized) as well as pathogen-associated traits between the different lineages within *Pantoea*. Despite the association of various *Pantoea* species with only clinical infections (*P. brenneri, P. conspicua*, and *P. eucrina*), it appears that species associated with opportunistic clinical infections (De Baere et al., 2004; Cruz et al., 2007), possessed genes often associated with animal pathogenicity within the *Enterobacteriaceae*. In general, it appears that the lineages with the most diverse niche associated processes also corresponded to those species isolated from the most diverse environments. For example, members of the *P. agglomerans* and *P. ananatis* lineages are routinely isolated from various plant species, as epiphytes, endophytes, or pathogens, as well as from insects, animals and humans (Coutinho and Venter, 2009; Walterson and Stavrinides, 2015), while members of the *P. dispersa* and *P. rodasii* lineages are usually only associated with a single host organism, with the exception of *P. dispersa*. These characteristics may contribute to the opportunistic nature of lineages containing species like *P. ananatis* and *P. agglomerans* that are proven plant pathogens, but isolated from diverse environments including the clinical setting.

### Evolution of multi-gene pathways in *Pantoea* and its lineages

Various evolutionary mechanisms likely shaped the presence and distribution of the multi-gene pathways inferred for *Pantoea*. Bacteria propagate asexually and progeny are anticipated to contain the same genetic material as the parent (Daubin et al., 2002). Any changes in an individual's genetic makeup can become fixed in populations if they provide a competitive advantage, or at least have no deleterious effects (Cohan, 2001, 2005; Caro-Quintero and Konstantinidis, 2012). The most common forces facilitating genetic change are random mutations (point mutations as well as insertions and deletions) and horizontal gene transfer (HGT) (Gogarten et al., 2002; Cohen et al., 2011). Accordingly, more closely related species are likely to encode similar pathways, while those subject to HGT would have a more spurious distribution (Gogarten et al., 2002; Cohen et al., 2011).

Evolution of most of the multi-gene pathways in *Pantoea* and its lineages involved complex processes, involving vertical descent with lineage- and species-specific gene losses/gains via duplication or HGT. For example, lineage- and species-specific gene losses would be characterized by distribution patterns where particular gene clusters are present in all taxa neighboring a species lacking it, because of gene losses at a specific ancestral node (Koskiniemi et al., 2012). In our study, such processes were likely involved in the loci required for the conversion of chloroacetaldehyde to glycolate and in the methionine salvage pathway. In contrast to this, the sudden appearance of genes or loci in a lineage that are lacking in all neighboring taxa suggest they were acquired via HGT (Zaneveld et al., 2008). An example of this is the locus required for protochatechuate utilization. The evolution of the siderophore loci likely involved gene losses together with the horizontal acquisition of genes. This is evident from the absence of the enterobactin locus (despite its presence in all closely related lineages) from the *P. ananatis* lineage, and the presence of the locus encoding aerobactin in only this lineage, thus acquired through an HGT event.

Our results suggested that the gene clusters encoding the various *Pantoea* pathways examined in this study, mainly experience purifying selection. Numerous hypotheses attempt to explain why genes cluster and how clusters are maintained (Carbone et al., 2007; Geddy and Brown, 2007; Fang et al., 2008; Sorrels et al., 2009). However, various studies have showed that purifying selection may contribute to the stability and functionality of gene clusters once they have formed (Carbone et al., 2007; Geddy and Brown, 2007; Fang et al., 2008; Sorrels et al., 2009). Purifying selection seems to facilitate the maintenance of ancient or ancestral gene clusters by limiting the possibility of non-synonymous mutations becoming fixed, which in turn allows out-competition of individuals undergoing deleterious or lethal mutations inhibiting the functioning of the gene cluster (Fang et al., 2008; Sorrels et al., 2009). In our study, purifying selection appeared to play a role in the maintenance and functioning of the loci for siderophore biosynthesis (aerobactin and enterobactin loci) and protochatechuate utilization, which are evolving mainly under the influence of purifying selection. However, we also detected diversifying selection for some of the genes/gene regions examined [e.g., *rfbC* and *rfbD* (*rfb* locus 1), both associated with the conversion of dTDP-6-deoxy-D-xylo-4-hexulose to dTDP-L-rhamnose (Graninger et al., 1999)]. In these cases, selection is causing non-synonymous changes in the sequences of genes or parts of genes, thus driving the appearance of new alleles.

### *In silico* predictions vs. experimentally confirmed multi-gene processes

Although a large number of differential characters were identified from the genome sequences of these organisms, little has been done in terms of experimental verification. Also, a number of characters could not be correlated to current experimental knowledge as false negative and positive results for phenotypic tests are common (Sutcliffe et al., 2012). For instance, despite the presence of genes required for the utilization of arbutin and salicin as carbon source in *Tatumella*, previous phenotypic tests have previously tested negative (Brady et al., 2010b; Tracz et al., 2015). This was also observed for the intra-generic comparisons. Examples within *Pantoea* included the uptake and utilization of cellobiose (Brady et al., 2009) and D-galacturonate, previously identified as a genus-defining attribute (Gavini et al., 1989; Brady et al., 2010a), as well as citrate (Brady et al., 2010a, 2012). In these cases, phenotypic tests previously confirmed the ability to perform these functions, but these genes were lacking in various *Pantoea* species (absence confirmed in available genome sequences of additional isolates of these species). This lack in correlation may either be as a result of gene expression complexities during phenotypic tests, to sequencing, assembly or annotation errors or the presence of as of yet uncharacterized alternate pathways.

There were, however, also a number of *in silico* functional predictions that correlated well with the results of previous phenotypic tests. For example, the lack of genes required for the utilization of histidine in *Tatumella*, correlated entirely with negative results of all previous phenotypic tests (Brady et al., 2010b; Tracz et al., 2015). Moreover, the utilization of protochatechuate has previously tested positive in *P. allii* (Brady et al., 2011), and our findings showed that the locus encoding the necessary gene products is indeed present within this lineage. Other examples of characters supporting previously performed phenotypic tests are the transport for the utilization of D-xylose, maltose, myo-inositol-1-P, and sucrose. In all of the above mentioned examples gene clusters were only observed in taxa previously testing positive for the associated phenotypic characters (Brady et al., 2010a,b, 2011).

### Perspectives and relevance

The increase in data obtained from bacterial genome sequences has superseded the rate at which gene discovery, characterization, and verification can occur. This means that a number of genes could not be assigned definitive functions as no homologs were detected for these genes in the KEGG database. The majority of these genes, at both the generic and lineage-specific level, could be annotated with gene ontologies with Blast2GO, although the functions of some genes remained unknown. Of the uncharacterized genes, the most abundant GO terms were generally quite similar (data for genera not shown). This indicates that the unknown genes could be performing similar functions within the different lineages or genera, despite not being orthologous amongst the different gene sets. The genes to which no functional annotation could be assigned seemed to have originated mostly within the *Enterobacteriaceae*, either acquired through lateral acquisition of genes from other taxa within this family, or, in the case where genes are conserved in most of the lineages, acquired by an ancestor and subsequently lost in some lineages when the genes were no longer required. A number of unique genes were also identified with no known homologs, but expression of these genes should be confirmed before they are considered for further investigation. Although no functional information is available for the unannotated genes, the distribution across various taxa provides insight into potential HGT events.

The availability of whole genome sequence data has transformed the approaches available for understanding bacterial evolution. This has, however, not yet replaced traditional methods such as DNA-DNA hybridization, monophyly in phylogenetic analyses and physiological and metabolic tests for the delineation of bacterial taxa. Although physiologic capabilities provide an insight into what potential metabolic differentiation may have occurred during speciation, inconsistencies may still occur due to differential expression of genes in different isolates or regulation of expression in specific environments. In recent years, we have been moving toward approaches aiming to identify natural groups at higher taxonomic levels by implementing monophyly as a prerequisite for taxon descriptions, but no light can be shed on the biology of the organisms through these approaches. Instead we need to investigate the more variable genomic compartments reflecting the biology of the organisms to obtain a more natural and robust taxonomic system. By employing this approach, one would be able to supplement or supplant the available diagnostic characters used in bacterial taxonomy. Additionally, obtaining a holistic biological perspective from the genome will provide power to predict the lifestyle and ecology of the organisms and is essentially much more informative than only having discriminative power between taxa. We thus believe that this approach of identifying genome-based characteristics in metabolic networks for the taxonomic levels higher than the species, provide an approach of identifying biologically relevant differences along the course of speciation.

## Author contributions

All authors contributed toward the original concept or data analyses, together with the drafting, revision and approval of the final manuscript. MP, ES, MC, and SV: was involved in the conceptualization and design of the work; MP and JB: was involved in data acquisition and analysis and all authors were involved in the interpretation of data.

### Conflict of interest statement

The authors declare that the research was conducted in the absence of any commercial or financial relationships that could be construed as a potential conflict of interest.

## Supplementary material

Supplementary Table S1 Environmental information processing traits with differences among the lineages.

Supplementary File S1 Output from GhostKOALA for the *Pantoea* and *Tatumella* shared gene sets. This file contains the differences observed for the overview maps and the specific pathways for *Pantoea* and *Tatumella*. The overlay figures of the overview and pathway maps are also indicated.

Supplementary File S2 Output from GhostKOALA for the lineages within *Pantoea*. This file contains the differences observed for the overview maps as well as the overlay figures of the overview maps for the different lineages.

Supplementary File S3 The summary of differences in pathways requiring 2 or more genes, as well as a summary of the BLAST confirmations of these genes.

Supplementary File S4 The results from the selection analyses and the figures for the gene clusters not indicated in text.

Supplementary File S5 The differences for pathways involved in “Environmental Information Processing”. A summary of the BLAST confirmation is also included as well as the maps for each lineage for the ABC transporters, two-component systems and the PTSs.

Supplementary File S6 Summary of the Blast2GO analyses as well as the BLAST hits for genes not annotated with Blast2GO. A pie chart indicating the distribution of BLAST hits is also indicated.

## References

[B1] Albers E. (2009). Metabolic characteristics and importance of the universal methionine salvage pathway recycling methionine from 5′-methylthioadenosine. IUBMB Life 61, 1132–1142. 10.1002/iub.278 19946895

[B2] Altschul S. F. Gish W. Miller W. Myers E. W. Lipman D. J. (1990). Basic local alignment search tool. J. Mol. Biol. 215, 403–410. 10.1016/S0022-2836(05)80360-2 2231712

[B3] Amemura A. Chakraborty R. Fujita M. Noumi T. Futai M. (1988). Cloning and nucleotide sequence of the isoamylase gene from *Pseudomonas amyloderamosa* SB-15. J. Biol. Chem. 263, 9271–9275. 3379068

[B4] Anderson W. A. Magasanik B. (1971). The pathway of myo-inositol degradation in *Aerobacter aerogenes*: conversion of 2-deoxy-5-keto-d-gluconic acid to glycolytic intermediates. J. Biol. Chem. 246, 5662–5675. 4328832

[B5] Armenta-Medina D. Segovia L. Perez-Rueda E. (2014). Comparative genomics of nucleotide metabolism: a tour to the past of the three cellular domains of life. BMC Genomics 15:800. 10.1186/1471-2164-15-800 25230797 PMC4177761

[B6] Badía J. Ros J. Aguilar J. (1985). Fermentation mechanism of fucose and rhamnose in *Salmonella typhimurium* and *Klebsiella pneumoniae*. J. Bacteriol. 161, 435–437. 3918008 10.1128/jb.161.1.435-437.1985 PMC214891

[B7] Baecker P. A. Greenberg E. Preiss J. (1986). Biosynthesis of bacterial glycogen. Primary structure of *Escherichia coli* 1,4-alpha-D-glucan:1,4-alpha-D-glucan 6-alpha-D-(1, 4-alpha-D-glucano)-transferase as deduced from the nucleotide sequence of the glg B gene. J. Biol. Chem. 261, 8738–8743. 3013861

[B8] Bennett J. S. Jolley K. A. Earle S. G. Corton C. Bentley S. D. Parkhill J.. (2012). A genomic approach to bacterial taxonomy: an examination and proposed reclassification of species within the genus *Neisseria*. Microbiology. 158, 1570–1580. 10.1099/mic.0.056077-0 22422752 PMC3541776

[B9] Berkowitz D. (1971). D-Mannitol utilization in *Salmonella typhimurium*. J. Bacteriol. 105, 232–240. 4322346 10.1128/jb.105.1.232-240.1971 PMC248346

[B10] Beukes C. W. Palmer M. Manyaka P. Chan W. Y. Avontuur J. R. van Zyl E.. (2017). Genome Data provides high support for generic boundaries in burkholderia sensu lato. Front. Microbiol. 8:1154. 10.3389/fmicb.2017.01154 28694797 PMC5483467

[B11] Blom J. Kreis J. Spänig S. Juhre T. Bertelli C. Ernst C.. (2016). EDGAR 2.0: an enhanced software platform for comparative gene content analyses. Nucleic Acids Res. 44, W22–W28. 10.1093/nar/gkw255 27098043 PMC4987874

[B12] Boer H. ten Hoeve-Duurkens R. H. Robillard G. T. (1996). Relation between the oligomerization state and the transport and phosphorylation function of the *Escherichia coli* mannitol transport protein: interaction between mannitol-specific enzyme II monomers studied by complementation of inactive site-directed mutants. Biochemistry 35, 12901–12908. 10.1021/bi9611016 8841134

[B13] Brady C. L. Cleenwerck I. van der Westhuizen L. Venter S. N. Coutinho T. A. De Vos P. (2012). *Pantoea rodasii* sp. nov., *Pantoea rwandensis* sp. nov. and *Pantoea wallisii* sp. nov., isolated from *Eucalyptus*. Int. J. Syst. Evol. Microbiol. 62, 1457–1464. 10.1099/ijs.0.032615-0 21841003

[B14] Brady C. L. Cleenwerck I. Venter S. N. Engelbeen K. De Vos P. Coutinho T. A. (2010a). Emended description of the genus Pantoea, description of four species from human clinical samples, *Pantoea septica* sp. nov., *Pantoea eucrina* sp. nov., *Pantoea brenneri* sp. nov. and *Pantoea conspicua* sp. nov., and transfer of *Pectobacterium cypripedii* (Hori 1911) Brenner et al. 1973 emend. Hauben et al. 1998 to the genus as *Pantoea cypripedii* comb. nov. Int. J. Syst. Evol. Microbiol. 60, 2430–2440. 10.1099/ijs.0.017301-0 19946052

[B15] Brady C. L. Goszczynska T. Venter S. N. Cleenwerck I. De Vos P. Gitaitis R. D.. (2011). *Pantoea allii* sp. nov., isolated from onion plants and seed. Int. J. Syst. Evol. Microbiol. 61, 932–937. 10.1099/ijs.0.022921-0 20495023

[B16] Brady C. L. Venter S. N. Cleenwerck I. Engelbeen K. Vancanneyt M. Swings J.. (2009). *Pantoea vagans* sp. nov., *Pantoea eucalypti* sp. nov., *Pantoea deleyi* sp. nov. and *Pantoea anthophila* sp. nov. Int. J. Syst. Evol. Microbiol. 59, 2339–2345. 10.1099/ijs.0.009241-0 19620357

[B17] Brady C. L. Venter S. N. Cleenwerck I. Vandemeulebroecke K. De Vos P. Coutinho T. A. (2010b). Transfer of *Pantoea citrea, Pantoea punctata* and *Pantoea terrea* to the genus *Tatumella* emend. as *Tatumella citrea* comb. nov., *Tatumella punctata* comb. nov. and *Tatumella terrea* comb. nov. and description of *Tatumella morbirosei* sp. nov. Int. J. Syst. Evol. Microbiol. 60, 484–494. 10.1099/ijs.0.012070-0 19654354

[B18] Brady C. Cleenwerck I. Venter S. Coutinho T. De Vos P. (2013). Taxonomic evaluation of the genus Enterobacter based on multilocus sequence analysis (MLSA): proposal to reclassify E. nimipressuralis and *E. amnigenus* into *Lelliottia* gen. nov. as *Lelliottia nimipressuralis* comb. nov. and *Lelliottia amnigena* comb. nov., respectively, *E. gergoviae* and *E. pyrinus* into *Pluralibacter* gen. nov. as *Pluralibacter gergoviae* comb. nov. and *Pluralibacter pyrinus* comb. nov., respectively, *E. cowanii, E. radicincitans, E. oryzae* and *E. arachidis* into *Kosakonia* gen. nov. as *Kosakonia cowanii* comb. nov., *Kosakonia radicincitans* comb. nov., *Kosakonia oryzae* comb. nov. and *Kosakonia arachidis* comb. nov., respectively, and *E. turicensis, E. helveticus* and *E. pulveris* into *Cronobacter* as *Cronobacter zurichensis* nom. nov., *Cronobacter helveticus* comb. nov. and *Cronobacter pulveris* comb. nov., respectively, and emended description of the genera *Enterobacter* and Cronobacter. Syst. Appl. Microbiol. 36, 309–319. 10.1016/j.syapm.2013.03.005 23632228

[B19] Carbone I. Ramirez-Prado J. H. Jakobek J. L. Horn B. W. (2007). Gene duplication, modularity and adaptation in the evolution of the aflatoxin gene cluster. BMC Evol. Biol. 7:111. 10.1186/1471-2148-7-111 17620135 PMC1949824

[B20] Caro-Quintero A. Konstantinidis K. T. (2012). Bacterial species may exist, metagenomics reveal. Environ. Microbiol. 14, 347–355. 10.1111/j.1462-2920.2011.02668.x 22151572

[B21] Cendron L. Berni R. Folli C. Ramazzina I. Percudani R. Zanotti G. (2007). The structure of 2-Oxo-4-hydroxy-4-carboxy-5-ureidoimidazoline decarboxylase provides insights into the mechanism of uric acid degradation. J. Biol. Chem. 282, 18182–18189. 10.1074/jbc.M701297200 17428786

[B22] Chan J. Z. M. Halachev M. R. Loman N. J. Constantinidou C. Pallen M. J. (2012). Defining bacterial species in the genomic era: insights from the genus *Acinetobacter*. BMC Microbiol. 12:302. 10.1186/1471-2180-12-302 23259572 PMC3556118

[B23] Chandra G. Chater K. F. Bornemann S. (2011). Unexpected and widespread connections between bacterial glycogen and trehalose metabolism. Microbiology 157, 1565–1572. 10.1099/mic.0.044263-0 21474533

[B24] Cohan F. M. (2001). Bacterial species and speciation. Syst. Biol. 50, 513–524. 10.1080/10635150118398 12116650

[B25] Cohan F. M. (2002). What are bacterial species? Annu. Rev. Microbiol. 56, 457–487. 10.1146/annurev.micro.56.012302.160634 12142474

[B26] Cohan F. M. (2005). Periodic selection and ecological diversity in bacteria, in Selective Sweep, ed Nurminsky D. (Georgetown, TX: Springer), 78–93.

[B27] Cohen O. Gophna U. Pupko T. (2011). The complexity hypothesis revisited: connectivity rather than function constitutes a barrier to horizontal gene transfer. Mol. Biol. Evol. 28, 1481–1489. 10.1093/molbev/msq333 21149642

[B28] Colloc'h N. Hajji M. E. Bachet B. L'Hermite G. Schiltz M. Prange T.. (1997). Crystal structure of the protein drug urate oxidase-inhibitor complex at 2.05 A resolution. Nat. Struct. Mol. Biol. 4, 947–952. 10.1038/nsb1197-947 9360612

[B29] Conesa A. Götz S. García-Gómez J. M. Terol J. Talón M. Robles M. (2005). Blast2GO: a universal tool for annotation, visualization and analysis in functional genomics research. Bioinformatics 21, 3674–3676. 10.1093/bioinformatics/bti610 16081474

[B30] Coutinho T. A. Venter S. N. (2009). Pantoea ananatis: an unconventional plant pathogen. Mol. Plant Pathol. 10, 325–335. 10.1111/j.1364-3703.2009.00542.x 19400836 PMC6640510

[B31] Cruz A. T. Cazacu A. C. Allen C. H. (2007). *Pantoea agglomerans*, a plant pathogen causing human disease. J. Clin. Microbiol. 45, 1989–1992. 10.1128/JCM.00632-07 17442803 PMC1933083

[B32] Dahl U. Jaeger T. Nguyen B. T. Sattler J. M. Mayer C. (2004). Identification of a phosphotransferase system of *Escherichia coli* required for growth on N-Acetylmuramic acid. J. Bacteriol. 186, 2385–2392. 10.1128/JB.186.8.2385-2392.2004 15060041 PMC412175

[B33] Daubin V. Gouy M. Perrière G. (2002). A phylogenomic approach to bacterial phylogeny: evidence of a core of genes sharing a common history. Genome Res. 12, 1080–1090. 10.1101/gr.187002 12097345 PMC186629

[B34] De Baere T. Verhelst R. Labit C. Verschraegen G. Wauters G. Claeys G.. (2004). Bacteremic infection with *Pantoea ananatis*. J. Clin. Microbiol. 42, 4393–4395. 10.1128/JCM.42.9.4393-4395.2004 15365053 PMC516322

[B35] De Maayer P. Chan W. Y. Rezzonico F. Bühlmann A. Venter S. N. Blom J.. (2012a). Complete genome sequence of clinical isolate *Pantoea ananatis* LMG5342. J. Bacteriol. 194, 1615–1616. 10.1128/JB.06715-11 22374951 PMC3294857

[B36] De Maayer P. Chan W.-Y. Blom J. Venter S. N. Duffy B. Smits T. H. M.. (2012b). The large universal *Pantoea plasmid* LPP-1 plays a major role in biological and ecological diversification. BMC Genomics 13:625. 10.1186/1471-2164-13-625 23151240 PMC3505739

[B37] De Maayer P. Chan W.-Y. Rubagotti E. Venter S. N. Toth I. K. Birch P. R. J.. (2014). Analysis of the *Pantoea ananatis* pan-genome reveals factors underlying its ability to colonize and interact with plant, insect and vertebrate hosts. BMC Genomics 15:404. 10.1186/1471-2164-15-404 24884520 PMC4070556

[B38] Deeg R. Roder A. Siedel J. Gauhl H. Ziegenhorn J. Boehringer Mannheim Gmbh (1987). Process and Reagent for the Determination of N-Carbamoylsarcosine with the Use of a New Enzyme. U.S. Patent 4,645,739.

[B39] Fang G. Rocha E. P. Danchin A. (2008). Persistence drives gene clustering in bacterial genomes. BMC Genomics 9:4. 10.1186/1471-2164-9-4 18179692 PMC2234087

[B40] Fecteau G. Fairbrother J. M. Higgins R. Van Metre D. C. Par,é J. Smith B. P.. (2001). Virulence factors in *Escherichia coli* isolated from the blood of bacteremic neonatal calves. Vet. Microbiol. 78, 241–249. 10.1016/S0378-1135(00)00299-6 11165068

[B41] Fiedler H.-P. Krastel P. Müller J. Gebhardt K. Zeeck A. (2001). Enterobactin: the characteristic catecholate siderophore of Enterobacteriaceae is produced by *Streptomyces* species. FEMS Microbiol. Lett. 196, 147–151. 10.1111/j.1574-6968.2001.tb10556.x 11267771

[B42] Fouet A. Klier A. Rapoport G. (1986). Nucleotide sequence of the sucrase gene of *Bacillus subtilis*. Gene 45, 221–225. 10.1016/0378-1119(86)90258-1 3100393

[B43] Fujita Y. Matsuoka H. Hirooka K. (2007). Regulation of fatty acid metabolism in bacteria. Mol. Microbiol. 66, 829–839. 10.1111/j.1365-2958.2007.05947.x 17919287

[B44] Gao Q. Wang X. Xu H. Xu Y. Ling J. Zhang D.. (2012). Roles of iron acquisition systems in virulence of extraintestinal pathogenic *Escherichia coli*: salmochelin and aerobactin contribute more to virulence than heme in a chicken infection model. BMC Microbiol. 12:143. 10.1186/1471-2180-12-143 22817680 PMC3496646

[B45] Garcia E. C. Brumbaugh A. R. Mobley H. L. (2011). Redundancy and specificity of *Escherichia coli* iron acquisition systems during urinary tract infection. Infect. Immun. 79, 1225–1235. 10.1128/IAI.01222-10 21220482 PMC3067483

[B46] Garrity G. M. Bell J. A. Lilburn T. (2005). The revised road map to the *manual*, in Bergey's Manual® of Systematic Bacteriology, eds Brenner D. J. Krieg N. R. Staley J. R. Garrity G. M. (Boston, MA: Springer), 159–187.

[B47] Gavini F. Mergaert J. Beji A. Mielcarek C. Izard D. Kersters K. (1989). Transfer of *Enterobacter agglomerans* (Beijerinck 1888) Ewing and Fife 1972 to *Pantoea* gen. *nov*. as *Pantoea agglomerans* comb. nov. and description of *Pantoea dispersa* sp. nov. Int. J. Syst. Bacteriol. 39, 337–345. 10.1099/00207713-39-3-337

[B48] Geddy R. Brown G. G. (2007). Genes encoding pentatricopeptide repeat (PPR) proteins are not conserved in location in plant genomes and may be subject to diversifying selection. BMC Genomics 8:30 10.1186/1471-2164-8-130 17521445 PMC1892557

[B49] Gevers D. Cohan F. M. Lawrence J. G. Spratt B. G. Coenye T. Feil E. J.. (2005). Re-evaluating prokaryotic species. Nature Rev. 3, 733–739. 10.1038/nrmicro1236 16138101

[B50] Giorgelli F. Bottai C. Mascia L. Scolozzi C. Camici M. Ipata P. L. (1997). Recycling of α-d-ribose 1-phosphate for nucleoside interconversion. Biochim. Biophys. Acta 1335, 16–22. 10.1016/S0304-4165(96)00117-1 9133638

[B51] Glaeser S. P. Kämpfer P. (2015). Multilocus sequence analysis (MLSA) in prokaryotic taxonomy. Syst. Appl. Microbiol. 38, 237–245. 10.1016/j.syapm.2015.03.007 25959541

[B52] Gogarten J. P. Doolittle W. F. Lawrence J. G. (2002). Prokaryotic evolution in light of gene transfer. Mol. Biol. Evol. 19, 2226–2238. 10.1093/oxfordjournals.molbev.a004046 12446813

[B53] Gossmann T. I. Ziegler M. Puntervoll P. de Figueiredo L. F. Schuster S. Heiland I. (2012). NAD+ biosynthesis and salvage–a phylogenetic perspective. FEBS J. 279, 3355–3363. 10.1111/j.1742-4658.2012.08559.x 22404877

[B54] Götz S. García-Gómez J. M. Terol J. Williams T. D. Nagaraj S. H. Nueda M. J.. (2008). High-throughput functional annotation and data mining with the Blast2GO suite. Nucleic Acids Res. 36, 3420–3435. 10.1093/nar/gkn176 18445632 PMC2425479

[B55] Gowrishankar J. (1989). Nucleotide sequence of the osmoregulatory proU operon of *Escherichia coli*. J. Bacteriol. 171, 1923–1931. 10.1128/jb.171.4.1923-1931.1989 2649479 PMC209841

[B56] Graninger M. Nidetzky B. Heinrichs D. E. Whitfield C. Messner P. (1999). Characterization of dTDP-4-dehydrorhamnose 3,5-Epimerase and dTDP-4-dehydrorhamnose reductase, required for dTDP-l-rhamnose biosynthesis in *Salmonella enterica* S*erovar Typhimurium* LT2. J. Biol. Chem. 274, 25069–25077. 10.1074/jbc.274.35.25069 10455186

[B57] Greenfield L. K. Whitfield C. (2012). Synthesis of lipopolysaccharide O-antigens by ABC transporter-dependent pathways. Carbohydr. Res. 356, 12–24. 10.1016/j.carres.2012.02.027 22475157

[B58] Gueule D. Fourny G. Ageron E. Le Flèche-Matéos A. Vandenbogaert M. Grimont P. A. D.. (2015). *Pantoea coffeiphila* sp. nov., cause of the ‘potato taste’ of Arabica coffee from the African Great Lakes region. Int. J. Syst. Evol. Microbiol. 65, 23–29. 10.1099/ijs.0.063545-0 25267869

[B59] Gugliucci A. Menini T. (2003). The polyamines spermine and spermidine protect proteins from structural and functional damage by AGE precursors: a new role for old molecules? Life Sci. 72, 2603–2616. 10.1016/S0024-3205(03)00166-8 12672506

[B60] Ha H. C. Sirisoma N. S. Kuppusamy P. Zweier J. L. Woster P. M. Casero R. A. (1998). The natural polyamine spermine functions directly as a free radical scavenger. Proc. Natl. Acad. Sci. U.S.A. 95, 11140–11145. 10.1073/pnas.95.19.11140 9736703 PMC21609

[B61] Hacker J. Carniel E. (2001). Ecological fitness, genomic islands and bacterial pathogenicity. EMBO Rep. 2, 376–381. 10.1093/embo-reports/kve097 11375927 PMC1083891

[B62] Hall B. G. Xu L. (1992). Nucleotide sequence, function, activation, and evolution of the cryptic asc operon of *Escherichia coli* K12. Mol. Biol. Evol. 9, 688–706. 1630307 10.1093/oxfordjournals.molbev.a040753

[B63] Hématy K. Cherk C. Somerville S. (2009). Host–pathogen warfare at the plant cell wall. Curr. Opin. Plant Biol. 12, 406–413. 10.1016/j.pbi.2009.06.007 19616468

[B64] Hillis D. M. Dixon M. T. (1991). Ribosomal DNA: molecular evolution and phylogenetic inference. Q. Rev. Biol. 66, 411–453. 10.1086/417338 1784710

[B65] Hong K.-W. Gan H. M. Low S.-M. Lee P. K. Y. Chong Y.-M. Yin W.-F.. (2012). Draft genome sequence of *Pantoea* sp. strain A4, a Rafflesia-associated bacterium that produces N-acylhomoserine lactones as quorum-sensing molecules. J. Bacteriol. 194:6610. 10.1128/JB.01619-12 23144374 PMC3497480

[B66] Hosie A. H. F. Poole P. S. (2001). Bacterial ABC transporters of amino acids. Res. Microbiol. 152, 259–270. 10.1016/S0923-2508(01)01197-4 11421273

[B67] Hubertus H. Schoeser M. Lesuisse E. Ernst J. F. Parson W. Beate A. (2003). Characterization of the *Aspergillus nidulans* transporters for the siderophores enterobactin and triacetylfusarinine C. Biochem. J. 371, 505–513. 10.1042/bj20021685 12487628 PMC1223275

[B68] Jaeger T. Mayer C. (2008). N-acetylmuramic acid 6-phosphate lyases (MurNAc etherases): role in cell wall metabolism, distribution, structure, and mechanism. Cell. Mol. Life Sci. 65, 928–939. 10.1007/s00018-007-7399-x 18049859 PMC11131651

[B69] Javaux C. Joris B. De Witte P. (2007). Functional Characteristics of TauA binding protein from TauABC *Escherichia coli* system. Protein J. 26, 231–238. 10.1007/s10930-006-9064-x 17203388

[B70] Jones P. Binns D. Chang H.-Y. Fraser M. Li W. McAnulla C.. (2014). InterProScan 5: genome-scale protein function classification. Bioinformatics 30, 1236–1240. 10.1093/bioinformatics/btu031 24451626 PMC3998142

[B71] Kamber T. Smits T. H. M. Rezzonico F. Duffy B. (2012). Genomics and current genetic understanding of *Erwinia amylovora* and the fire blight antagonist *Pantoea vagans*. Trees 26, 227–238. 10.1007/s00468-011-0619-x

[B72] Kanehisa M. Goto S. Kawashima S. Nakaya A. (2002). The KEGG databases at GenomeNet. Nucleic Acids Res. 30, 42–46. 10.1093/nar/30.1.42 11752249 PMC99091

[B73] Kanehisa M. Sato Y. Morishima K. (2016a). BlastKOALA and GhostKOALA: KEGG tools for functional characterization of genome and metagenome sequences. J. Mol. Biol. 428, 726–731. 10.1016/j.jmb.2015.11.006 26585406

[B74] Kanehisa M. Sato Y. Kawashima M. Furumichi M. Tanabe M. (2016b). KEGG as a reference resource for gene and protein annotation. Nucleic Acids Res. 44, D457–D462. 10.1093/nar/gkv1070 26476454 PMC4702792

[B75] Kappes R. M. Kempf B. Kneip S. Boch J. Gade J. Meier-Wagner J.. (1999). Two evolutionarily closely related ABC transporters mediate the uptake of choline for synthesis of the osmoprotectant glycine betaine in *Bacillus subtilis*. Mol. Microbiol. 32, 203–216. 10.1046/j.1365-2958.1999.01354.x 10216873

[B76] Katoh A. Uenohara K. Akita M. Hashimoto T. (2006). Early steps in the biosynthesis of NAD in arabidopsis start with aspartate and occur in the plastid. Plant Physiol. 141, 851–857. 10.1104/pp.106.081091 16698895 PMC1489895

[B77] Katoh K. Standley D. M. (2013). MAFFT multiple sequence alignment software version 7: improvements in performance and usability. Mol. Biol. Evol. 30, 772–780. 10.1093/molbev/mst010 23329690 PMC3603318

[B78] Kehres D. Hogg R. (1992). *Escherichia coli* K12 arabinose-binding protein mutants with altered transport properties. Protein Sci. 1, 1652–1660. 10.1002/pro.5560011213 1304895 PMC2142127

[B79] Kempf B. Bremer E. (1995). OpuA, an osmotically regulated binding protein-dependent transport system for the osmoprotectant glycine betaine in *Bacillus subtilis*. J. Biol. Chem. 270, 16701–16713. 10.1074/jbc.270.28.16701 7622480

[B80] Khan N. A. Quemener V. Moulinoux J.-P. (1992). Phorbol esters augment polyamine transport by influencing Na^+^-K^+^ pump in murine leukemia cells. Exp. Cell Res. 199, 378–382. 10.1016/0014-4827(92)90448-H 1312010

[B81] Kimiyoshi I. Yoshihiro A. Kumi N. Shinsei M. Tatsuo H. Osamu S. (1993). Cloning of the cDNA encoding human xanthine dehydrogenase (oxidase): structural analysis of the protein and chromosomal location of the gene. Gene 133, 279–284. 10.1016/0378-1119(93)90652-J 8224915

[B82] Ko R. Smith L. T. (1999). Identification of an ATP-Driven, osmoregulated glycine betaine transport system in *Listeria monocytogenes*. Appl. Environ. Microbiol. 65, 4040–4048. 10473414 10.1128/aem.65.9.4040-4048.1999 PMC99739

[B83] Kohchi C. Inagawa H. Nishizawa T. Yamaguchi T. Nagai S. Soma G.-I. (2006). Applications of lipopolysaccharide derived from *Pantoea agglomerans* (IP-PA1) for health care based on macrophage network theory. J. Biosci. Bioeng. 102, 485–496. 10.1263/jbb.102.485 17270712

[B84] Konstantinidis K. T. Tiedje J. M. (2005a). Genomic insights that advance the species definition for prokaryotes. Proc. Natl. Acad. Sci. U.S.A. 102, 2567–2572. 10.1073/pnas.0409727102 15701695 PMC549018

[B85] Konstantinidis K. T. Tiedje J. M. (2005b). Towards a genome-based taxonomy for prokaryotes. J. Bacteriol. 187, 6258–6264. 10.1128/JB.187.18.6258-6264.2005 16159757 PMC1236649

[B86] Konstantinidis K. T. Tiedje J. M. (2007). Prokaryotic taxonomy and phylogeny in the genomic era: advancements and challenges ahead. Curr. Opin. Microbiol. 10, 504–509. 10.1016/j.mib.2007.08.006 17923431

[B87] Koskiniemi S. Sun S. Berg O. G. Andersson D. I. (2012). Selection-driven gene loss in bacteria. PLoS Genet. 8:e1002787. 10.1371/journal.pgen.1002787 22761588 PMC3386194

[B88] Krismer B. Liebeke M. Janek D. Nega M. Rautenberg M. Hornig G.. (2014). Nutrient limitation governs *Staphylococcus aureus* metabolism and niche adaptation in the human nose. PLoS Pathog. 10:e1003862. 10.1371/journal.ppat.1003862 24453967 PMC3894218

[B89] Linghu B. Snitkin E. S. Holloway D. T. Gustafson A. M. Xia Y. DeLisi C. (2008). High-precision high-coverage functional inference from integrated data sources. BMC Bioinformatics 9:119. 10.1186/1471-2105-9-119 18298847 PMC2292694

[B90] Liu T. Vora H. Khosla C. (2010). Quantitative analysis and engineering of fatty acid biosynthesis in E. coli. Metab. Eng. 12, 378–386. 10.1016/j.ymben.2010.02.003 20184964

[B91] Ma Y. Yin Y. Rong C. Chen S. Liu Y. Wang S.. (2016). *Pantoea pleuroti* sp. nov., Isolated from the fruiting bodies of *Pleurotus eryngii*. Curr. Microbiol. 72, 207–212. 10.1007/s00284-015-0940-5 26581526

[B92] Maciejewska A. M. Ruszel K. P. Nieminuszczy J. Lewicka J. Sokołowska B. Grzesiuk E.. (2010). Chloroacetaldehyde-induced mutagenesis in *Escherichia coli*: the role of AlkB protein in repair of 3, N 4-ethenocytosine and 3, N 4-α-hydroxyethanocytosine. Mutat. Res. Fundamental Mol. Mech. Mutagenesis 684, 24–34. 10.1016/j.mrfmmm.2009.11.005 19941873

[B93] McInerney J. O. Pisani D. Bapteste E. O'Connell M. J. (2011). The public goods hypothesis for the evolution of life on earth. Biol. Direct 6:41. 10.1186/1745-6150-6-41 21861918 PMC3179745

[B94] Mehler A. H. Tabor H. (1953). Deamination of histidine to form urocanic acid In liver. J. Biol. Chem. 201, 775–784. 13061415

[B95] Mergaert J. Verdonck L. Kersters K. (1993). Transfer of *Erwinia ananas* (synonym, *Erwinia uredovora*) and *Erwinia stewartii* to the genus *Pantoea emend*. as *Pantoea ananas* (Serrano 1928) comb. nov. and *Pantoea stewartii* (Smith 1898) comb. nov., respectively, and description of *Pantoea stewartii* subsp. indologenes subsp. nov. Int. J. Syst. Bacteriol. 43, 162–173. 10.1099/00207713-43-1-162

[B96] Metlitskaya A. Kazakov T. Vondenhoff G. H. Novikova M. Shashkov A. Zatsepin T.. (2009). Maturation of the translation inhibitor Microcin C. J. Bacteriol. 191, 2380–2387. 10.1128/JB.00999-08 19168611 PMC2655510

[B97] Moffatt B. A. Ashihara H. (2002). Purine and pyrimidine nucleotide synthesis and metabolism. Arabidopsis Book 1:e0018. 10.1199/tab.0018 22303196 PMC3243375

[B98] Montgomerie J. Bindereif A. Neilands J. Kalmanson G. Guze L. (1984). Association of hydroxamate siderophore (aerobactin) with Escherichia coli isolated from patients with bacteremia. Infect. Immun. 46, 835–838. 6389366 10.1128/iai.46.3.835-838.1984 PMC261622

[B99] Moralejo P. Egan S. Hidalgo E. Aguilar J. (1993). Sequencing and characterization of a gene cluster encoding the enzymes for L-rhamnose metabolism in *Escherichia coli*. J. Bacteriol. 175, 5585–5594. 10.1128/jb.175.17.5585-5594.1993 8396120 PMC206615

[B100] Nohno T. Saito T. Hong J.-S. (1986). Cloning and complete nucleotide sequence of the *Escherichia coli* glutamine permease operon (glnHPQ). Mol. Gen. Genet. 205, 260–269. 10.1007/BF00430437 3027504

[B101] Novikova M. Metlitskaya A. Datsenko K. Kazakov T. Kazakov A. Wanner B.. (2007). The *Escherichia coli* Yej transporter is required for the uptake of translation inhibitor Microcin C. J. Bacteriol. 189, 8361–8365. 10.1128/JB.01028-07 17873039 PMC2168686

[B102] Nygaard P. Bested S. M. Andersen K. A. K. Saxild H. H. (2000). *Bacillus subtilis* guanine deaminase is encoded by the yknA gene and is induced during growth with purines as the nitrogen source. Microbiology 146, 3061–3069. 10.1099/00221287-146-12-3061 11101664

[B103] Ollagnier-de Choudens S. Loiseau L. Sanakis Y. Barras F. Fontecave M. (2005). Quinolinate synthetase, an iron–sulfur enzyme in NAD biosynthesis. FEBS Lett. 579, 3737–3743. 10.1016/j.febslet.2005.05.065 15967443

[B104] Opal S. M. Cross A. S. Gemski P. Lyhte L. W. (1990). Aerobactin and α-hemolysin as virulence determinants in *Escherichia coli* isolated from human blood, urine, and stool. J. Infect. Dis. 161, 794–796. 10.1093/infdis/161.4.794 2181035

[B105] Ormeno-Orrillo E. Servín-Garcidue-as L. E. Rogel M. A. González V. Peralta H. Mora J.. (2015). Taxonomy of rhizobia and agrobacteria from the Rhizobiaceae family in light of genomics. Syst. Appl. Microbiol. 38, 287–291. 10.1016/j.syapm.2014.12.002 25660942

[B106] Palmer M. de Maayer P. Poulsen M. Steenkamp E. T. van Zyl E. Coutinho T. A.. (2016). Draft genome sequences of *Pantoea agglomerans* and *Pantoea vagans* isolates associated with termites. Stand. Genomic Sci. 11, 1–11. 10.1186/s40793-016-0144-z 26937267 PMC4774006

[B107] Palmer M. Steenkamp E. T. Coetzee M. P. A. Chan W.-Y. van Zyl E. De Maayer P.. (2017). Phylogenomic resolution of the bacterial genus *Pantoea* and its relationship with *Erwini*a and *Tatumella*. Antonie van Leeuwenhoek 110, 1287–1309. 10.1007/s10482-017-0852-4 28255640

[B108] Pegg A. E. (2016). Functions of polyamines in mammals. J. Biol. Chem. 291, 14904–14912. 10.1074/jbc.R116.731661 27268251 PMC4946908

[B109] Plumbridge J. (2009). An alternative route for recycling of N-Acetylglucosamine from peptidoglycan involves the N-Acetylglucosamine phosphotransferase system in *Escherichia coli*. J. Bacteriol. 191, 5641–5647. 10.1128/JB.00448-09 19617367 PMC2737974

[B110] Pond S. L. K. Muse S. V. (2005). HyPhy: hypothesis testing using phylogenies, in Statistical Methods in Molecular Evolution, ed Nielsen R. (New York, NY: Springer New York), 125–181.

[B111] Rahman N. A. Parks D. H. Vanwonterghem I. Morrison M. Tyson G. W. Hugenholtz P. (2015). A phylogenomic analysis of the bacterial phylum Fibrobacteres. Front. Microbiol. 6:1469. 10.3389/fmicb.2015.01469 26779135 PMC4704652

[B112] Raymond K. N. Dertz E. A. Kim S. S. (2003). Enterobactin: an archetype for microbial iron transport. Proc. Natl. Acad. Sci. U.S.A. 100, 3584–3588. 10.1073/pnas.0630018100 12655062 PMC152965

[B113] Richard P. Hilditch S. (2009). D-galacturonic acid catabolism in microorganisms and its biotechnological relevance. Appl. Microbiol. Biotechnol. 82, 597–604. 10.1007/s00253-009-1870-6 19159926

[B114] Richards V. P. Palmer S. R. Pavinski Bitar P. D. Qin X. Weinstock G. M. Highlander S. K.. (2014). Phylogenomics and the dynamic genome evolution of the genus *Streptococcus*. Genome Biol. Evol. 6, 741–753. 10.1093/gbe/evu048 24625962 PMC4007547

[B115] Rong C. Ma Y. Wang S. Liu Y. Chen S. Huang B.. (2016). *Pantoea hericii* sp. nov., isolated from the fruiting bodies of *Hericium erinaceus*. Curr. Microbiol. 72, 738–743. 10.1007/s00284-016-1011-2 26897127

[B116] Saxena R. Anand P. Saran S. Isar J. Agarwal L. (2010). Microbial production and applications of 1, 2-propanediol. Indian J. Microbiol. 50, 2–11. 10.1007/s12088-010-0017-x 23100801 PMC3450292

[B117] Schneider E. (2001). ABC transporters catalyzing carbohydrate uptake. Res. Microbiol. 152, 303–310. 10.1016/S0923-2508(01)01201-3 11421277

[B118] Schubert R. H. (1968). The taxonomy and nomenclature of the genus Aeromonas Kluyver and van Niel 1936. Int. J. Syst. Evol. Microbiol. 18, 1–7.

[B119] Schulz H. (1991). Beta oxidation of fatty acids. Biochimica Biophys. Acta 1081, 109–120. 10.1016/0005-2760(91)90015-A 1998729

[B120] Schwartz A. R. Potnis N. Timilsina S. Wilson M. Patané J. Martins J.. (2015). Phylogenomics of Xanthomonas field strains infecting pepper and tomato reveals diversity in effector repertoires and identifies determinants of host specificity. Front. Microbiol. 6:535. 10.3389/fmicb.2015.00535 26089818 PMC4452888

[B121] Schwede T. F. Rétey J. Schulz G. E. (1999). Crystal structure of histidine ammonia-lyase revealing a novel polypeptide modification as the catalytic electrophile. Biochemistry 38, 5355–5361. 10.1021/bi982929q 10220322

[B122] Scripture J. B. Voelker C. Miller S. O'Donnell R. T. Polgar L. Rade J.. (1987). High-affinity l-arabinose transport operon. J. Mol. Biol. 197, 37–46. 10.1016/0022-2836(87)90607-3 2445996

[B123] Sekowska A. Dénervaud V. Ashida H. Michoud K. Haas D. Yokota A.. (2004). Bacterial variations on the methionine salvage pathway. BMC Microbiol. 4:9. 10.1186/1471-2180-4-9 15102328 PMC395828

[B124] Sekowska A. Kung H.-F. Danchin A. (2000). Sulfur metabolism in *Escherichia coli* and related bacteria: facts and fiction. J. Mol. Microbiol. Biotechnol. 2, 145–177. 10939241

[B125] Shah P. Swiatlo E. (2008). A multifaceted role for polyamines in bacterial pathogens. Mol. Microbiol. 68, 4–16. 10.1111/j.1365-2958.2008.06126.x 18405343

[B126] Shimizu K. (2014). Regulation systems of bacteria such as *Escherichia coli* in response to nutrient limitation and environmental stresses. Metabolites 4:1. 10.3390/metabo4010001 24958385 PMC4018673

[B127] Sim L. Quezada-Calvillo R. Sterchi E. E. Nichols B. L. Rose D. R. (2008). Human intestinal maltase–glucoamylase: crystal structure of the N-terminal catalytic subunit and basis of inhibition and substrate specificity. J. Mol. Biol. 375, 782–792. 10.1016/j.jmb.2007.10.069 18036614

[B128] Song Y.-J. (2009). Characterization of aromatic hydrocarbon degrading bacteria isolated from pine litter. Kor. J. Microbiol. Biotechnol. 37, 333–339.

[B129] Sorrels C. M. Proteau P. J. Gerwick W. H. (2009). Organization, evolution, and expression analysis of the biosynthetic gene cluster for scytonemin, a cyanobacterial UV-absorbing pigment. Appl. Environ. Microbiol. 75, 4861–4869. 10.1128/AEM.02508-08 19482954 PMC2708446

[B130] Sprenger G. A. (1993). Two open reading frames adjacent to the *Escherichia coli* K-12 transketolase (tkt) gene show high similarity to the mannitol phosphotransferase system enzymes from *Escherichia coli* and various Gram-positive bacteria. Biochim. Biophys. Acta 1158, 103–106. 10.1016/0304-4165(93)90103-F 8353127

[B131] Stanier R. Y. Van Niel C. (1941). The main outlines of bacterial classification. J. Bacteriol. 42:437. 16560462 10.1128/jb.42.4.437-466.1941 PMC374769

[B132] Stevenson G. Neal B. Liu D. Hobbs M. Packer N. H. Batley M.. (1994). Structure of the O antigen of *Escherichia coli* K-12 and the sequence of its rfb gene cluster. J. Bacteriol. 176, 4144–4156. 10.1128/jb.176.13.4144-4156.1994 7517391 PMC205614

[B133] Stirling D. Hulton C. Waddell L. Park S. Stewart G. Booth I.. (1989). Molecular characterization of the proU loci of *Salmonella typhimurium* and *Escherichia coli* encoding osmoregulated glycine betaine transport systems. Mol. Microbiol. 3, 1025–1038. 10.1111/j.1365-2958.1989.tb00253.x 2691838

[B134] Sutcliffe I. C. (2015). Challenging the anthropocentric emphasis on phenotypic testing in prokaryotic species descriptions: rip it up and start again. Front. Genet. 6:218. 10.3389/fgene.2015.00218 26136772 PMC4469894

[B135] Sutcliffe I. C. Trujillo M. E. Goodfellow M. (2012). A call to arms for systematists: revitalising the purpose and practises underpinning the description of novel microbial taxa. Antonie Van Leeuwenhoek 101, 13–20. 10.1007/s10482-011-9664-0 22038177

[B136] Suzuki M. (1991). U.S. Patent No. 5,047,329. Washington, DC: U.S. Patent and Trademark Office.

[B137] Suzuki H. Koyanagi T. Izuka S. Onishi A. Kumagai H. (2005). The yliA, -B, -C, and -D genes of *Escherichia coli* K-12 encode a novel glutathione importer with an ATP-binding cassette. J. Bacteriol. 187, 5861–5867. 10.1128/JB.187.17.5861-5867.2005 16109926 PMC1196167

[B138] Tamura K. Stecher G. Peterson D. Filipski A. Kumar S. (2013). MEGA6: Molecular Evolutionary Genetics Analysis Version 6.0. Mol. Biol. Evol. 30, 2725–2729. 10.1093/molbev/mst197 24132122 PMC3840312

[B139] Tindall B. J. Rosselló-Mora R. Busse H.-J. Ludwig W. Kämpfer P. (2010). Notes on the characterization of prokaryote strains for taxonomic purposes. Int. J. Syst. Evol. Microbiol. 60, 249–266. 10.1099/ijs.0.016949-0 19700448

[B140] Torres A. G. Redford P. Welch R. A. Payne S. M. (2001). TonB-dependent systems of uropathogenic *Escherichia coli*: aerobactin and heme transport and TonB are required for virulence in the mouse. Infect. Immun. 69, 6179–6185. 10.1128/IAI.69.10.6179-6185.2001 11553558 PMC98749

[B141] Tracz D. M. Gilmour M. W. Mabon P. Beniac D. R. Hoang L. Kibsey P.. (2015). *Tatumella saanichensis* sp. nov., isolated from a cystic fibrosis patient. Int. J. Syst. Evolut. Microbiol. 65, 1959–1966. 10.1099/ijs.0.000207 25807976

[B142] Uehara T. Suefuji K. Jaeger T. Mayer C. Park J. T. (2006). MurQ etherase is required by *Escherichia coli* in order to metabolize anhydro-N-acetylmuramic acid obtained either from the environment or from its own cell wall. J. Bacteriol. 188, 1660–1662. 10.1128/JB.188.4.1660-1662.2006 16452451 PMC1367226

[B143] Van Beers E. H. Büller H. A. Grand R. J. Einerhand A. W. C. Dekker J. (1995). Intestinal brush border glycohydrolases: structure, function, and development. Crit. Rev. Biochem. Mol. Biol. 30, 197–262. 10.3109/10409239509085143 7555019

[B144] Van der Ploeg J. Weiss M. A. Saller E. Nashimoto H. Saito N. Kertesz M. A.. (1996). Identification of sulfate starvation-regulated genes in *Escherichia coli*: a gene cluster involved in the utilization of taurine as a sulfur source. J. Bacteriol. 178, 5438–5446. 10.1128/jb.178.18.5438-5446.1996 8808933 PMC178364

[B145] Vanneste J. Cornish D. Yu J. Voyle M. (2001). The peptide antibiotic produced by *Pantoea agglomerans* Eh252 is a microcin, in IX International Workshop on Fire Blight (Napier), 285–290.

[B146] Walshaw D. L. Lowthorpe S. East A. Poole P. S. (1997). Distribution of a sub-class of bacterial ABC polar amino acid transporter and identification of an N-terminal region involved in solute specificity. FEBS Lett. 414, 397–401. 10.1016/S0014-5793(97)01023-5 9315727

[B147] Walterson A. M. Stavrinides J. (2015). Pantoea: insights into a highly versatile and diverse genus within the Enterobacteriaceae. FEMS Microbiol. Rev. 39, 968–984. 10.1093/femsre/fuv027 26109597

[B148] Walton J. D. (1994). Deconstructing the cell wall. Plant Physiol. 104:1113. 10.1104/pp.104.4.1113 12232152 PMC159271

[B149] Wan X. Hou S. Phan N. Malone Moss J. S. Donachie S. P. Alam M. (2015). Draft genome sequence of pantoea anthophila strain 11-2 from hypersaline lake laysan, Hawaii. Genome Announc. 3:e00321–15. 10.1128/genomeA.00321-15 25977417 PMC4432323

[B150] Wang L. Reeves P. R. (1998). Organization of *Escherichia coli* O157 O antigen gene cluster and identification of its specific genes. Infect. Immun. 66, 3545–3551. 9673232 10.1128/iai.66.8.3545-3551.1998 PMC108385

[B151] Whitfield C. (1995). Biosynthesis of lipopolysaccharide O antigens. Trends Microbiol. 3, 178–185. 10.1016/S0966-842X(00)88917-9 7542987

[B152] Williams P. H. Carbonetti N. H. (1986). Iron, siderophores, and the pursuit of virulence: independence of the aerobactin and enterochelin iron uptake systems in *Escherichia coli*. Infect. Immun. 51, 942–947. 2936686 10.1128/iai.51.3.942-947.1986 PMC260990

[B153] Woese C. (1998). The universal ancestor. Proc. Natl. Acad. Sci. U.S.A. 95, 6854–6859. 10.1073/pnas.95.12.6854 9618502 PMC22660

[B154] Woese C. R. (1987). Bacterial evolution. Microbiol. Rev. 51:221. 2439888 10.1128/mr.51.2.221-271.1987 PMC373105

[B155] Woese C. R. (1994). There must be a prokaryote somewhere: microbiology's search for itself. Microbiol. Rev. 58, 1–9. 8177167 10.1128/mr.58.1.1-9.1994 PMC372949

[B156] Woese C. R. Kandler O. Wheelis M. L. (1990). Towards a natural system of organisms: proposal for the domains Archaea, Bacteria, and Eucarya. Proc. Natl. Acad. Sci. U.S.A. 87, 4576–4579. 10.1073/pnas.87.12.4576 2112744 PMC54159

[B157] Yang Q. Liu Y. Huang F. He Z.-G. (2011). Physical and functional interaction between d-ribokinase and topoisomerase I has opposite effects on their respective activity in *Mycobacterium smegmatis* and *Mycobacterium tuberculosis*. Arch. Biochem. Biophys. 512, 135–142. 10.1016/j.abb.2011.05.018 21683681

[B158] Yang Z. Savchenko A. Yakunin A. Zhang R. Edwards A. Arrowsmith C.. (2003). Aspartate dehydrogenase, a novel enzyme identified from structural and functional studies of TM1643. J. Biol. Chem. 278, 8804–8808. 10.1074/jbc.M211892200 12496312

[B159] Yoshida K.-I. Yamaguchi M. Ikeda H. Omae K. Tsurusaki K.-I. Fujita Y. (2004). The fifth gene of the iol operon of *Bacillus subtilis*, iolE, encodes 2-keto-myo-inositol dehydratase. Microbiology 150, 571–580. 10.1099/mic.0.26768-0 14993306

[B160] Yoshimoto T. Oka I. Tsuru D. (1976). Purification, crystallization, and some properties of creatine amidinohydrolase from *Pseudomonus putida*. J. Biochem. 79, 1381–1383. 10.1093/oxfordjournals.jbchem.a131193 8443

[B161] Young Kim M. Zhou X. Delaney J. C. Taghizadeh K. Dedon P. C. Essigmann J. M. (2007). AlkB influences the chloroacetaldehyde-induced mutation spectra and toxicity in the pSP189 supF shuttle vector. Chem. Res. Toxicol. 20, 1075–1083. 10.1021/tx700167v 17658757

[B162] Zaneveld J. R. Nemergut D. R. Knight R. (2008). Are all horizontal gene transfers created equal? Prospects for mechanism-based studies of HGT patterns. Microbiology 154, 1–15. 10.1099/mic.0.2007/011833-0 18174121

[B163] Zangoui P. Vashishtha K. Mahadevan S. (2015). Evolution of aromatic β-glucoside utilization by successive mutational steps in *Escherichia coli*. J. Bacteriol. 197, 710–716. 10.1128/JB.02185-14 25448815 PMC4334193

[B164] Zhang Z.-G. Ye Z.-Q. Yu L. Shi P. (2011). Phylogenomic reconstruction of lactic acid bacteria: an update. BMC Evol. Biol. 11:1. 10.1186/1471-2148-11-1 21194491 PMC3024227

