# Supplementary material for: Genome-Based Characterization of Biological Processes That Differentiate Closely Related Bacteria
Source: Front Microbiol. 2018 Feb 6;9:113. doi: 10.3389/fmicb.2018.00113 (PMC5808187; doi:10.3389/fmicb.2018.00113)
Supplement: Supplementary Table S1 — Environmental information processing traits with differences among the lineages. [file Table1.DOCX]

| **System Type** | **System** | **Gene cluster** | **Biological role** |
| --- | --- | --- | --- |
| Two-component systems | Citrate | *citAB* | Activates the signal transduction pathway for citrate utilization |
|  | Nitrate/ nitrite | *narPQ* | Activates the signal transduction pathway for nitrate/nitrite uptake |
| ABC Transporters | Nitrate/ nitrite/ cyanate | *nrtABC* | Transports of nitrate/nitrite/cyanate into cell using ATP |
|  | HMP/ FAMP | *thiXYZ* | Transports extracellular FAMP or HMP into cell using ATP for thiamine production |
|  | Spermidine/ Putrescine | *potABCD* | Transports spermidine or putrescine into cell using ATP |
|  | Putrescine | *potFGHI* | Transports putrescine into cell using ATP |
|  | Maltose/ Maltodextrin | *malEFGK* | Transports maltose/maltodextrin into cell using ATP for catabolism of compound |
|  | D-Xylose | *xylFGH* | Transports D-xylose into cell using ATP for catabolism of compound |
|  | Myoinositol-1-phosphate | *inoEFG* | Transports myoinositol-1-phosphate into cell using ATP for catabolism of compound |
|  | Phosphonate | *phnCDE* | Transports phosphonate into cell using ATP for cellular functions |
|  | Glutamine | *glnHPQ* | Transports glutamine into cell using ATP for utilization of amino acid |
|  | Arginine | *artIJMPQ* | Transports arginine into cell using ATP for utilization of amino acid |
|  | Urea | *urtABCDE* | Transports urea into cell using ATP for catabolism of compound |
|  | Glutathione | *gsiABCD* | Transports glutathione into cell using ATP for catabolism of compound |
|  | Iron (II)/ Manganese | *sitACD* | Transports iron(II) and manganese into cell using ATP for cellular functions |
| Phospho-transferase systems (PTSs) | Cellobiose | *celABC* | Phosphorylates and transfers cellobiose into cell for the catabolism of the compound |
|  | L-Ascorbate | *ulaABC* | Phosphorylates and transfers L-ascorbate into cell for the catabolism of the compound |

**Supplementary Table S1** Environmental information processing traits with differences among the lineages
